# Immediate effects of sitting position on cervical and trunk posture, perceived discomfort, and RULA-based ergonomic risk during smartphone gaming: A randomized within-subject study

**DOI:** 10.1371/journal.pone.0354360

**Published:** 2026-07-21

**Authors:** Patcharin Nilmart, Wantanee Yodchaisarn, Natdanai Vajidee, Nounchanok Tansakul, Wirada Sramongkhol, Sasisom Leangsakul

**Affiliations:** 1 Faculty of Physical Therapy, Srinakharinwirot University, Ongkharak, Nakhon Nayok, Thailand; 2 Department of Physical Therapy, School of Allied Health Sciences, Walailak University, Nakhon Si Thammarat, Thailand; 3 Movement Science and Exercise Research Center-Walailak University (MoveSE-WU), Nakhon Si Thammarat, Thailand; Prince Sattam bin Abdulaziz University, SAUDI ARABIA

## Abstract

**Objective:**

This study investigated the immediate effects of three sitting positions, including chair sitting with and without a backrest and cross-legged sitting on the floor, on cervical and trunk postures, musculoskeletal discomfort, and ergonomic risk during smartphone gaming among young adults.

**Methods:**

A randomized within-subject study was conducted in 41 healthy participants (mean age 20.4 ± 1.1 years). Each participant played Realm of Valor (ROV) on their personal smartphone for 20 minutes in three randomly assigned sitting positions. Cervical and trunk angles were measured using two-dimensional photogrammetry at baseline and at 5, 10, 15, and 20 minutes. Ergonomic risk was assessed using the Rapid Upper Limb Assessment (RULA), with the maximum score used for analysis. After each session, participants reported perceived musculoskeletal discomfort using a visual analog scale.

**Results:**

A significant interaction between time and sitting position was found for both neck flexion (F(3.87, 232.27) = 5.31, p < 0.001, partial η² = 0.081) and trunk angles (F(8,480) = 2.73, p = 0.006, partial η² = 0.044). Perceived neck discomfort was the most common symptom, with the cross-legged position showing the highest prevalence (51.2%, Cochran’s Q = 8.67, p = 0.013, Kendall’s W = 0.106). However, symptom intensity did not differ significantly among positions. RULA scores also differed across sitting positions (χ²(2) = 27.0, p < 0.001, Kendall’s W = 0.329), with the cross-legged position showing the highest score.

**Conclusion:**

Sitting posture significantly affected cervical and trunk alignment, perceived discomfort, and ergonomic risk during smartphone gaming. Cross-legged sitting induced the greatest neck and trunk flexion, which may contribute to increased musculoskeletal strain over time. These findings emphasize the importance of promoting ergonomic awareness and healthy digital habits to maintain spinal alignment and reduce posture-related musculoskeletal strain; however, longitudinal studies are needed to confirm long-term effects.

## Introduction

Smartphone use has become an integral part of daily life for communication, work, education, and entertainment, including competitive online gaming [1]. In recent years, smartphone ownership and daily screen time have increased substantially, contributing to more sedentary behaviors and even smartphone addiction [2]. These behaviors are often associated with flexed‑neck postures commonly described as “text neck,” characterized by prolonged cervical flexion while viewing the smartphone screen [3, 4]. The popularity of mobile gaming further amplifies these risks, as extended gameplay sessions may reinforce poor postural habits and contribute musculoskeletal discomfort, representing a growing public health concern.

Sitting posture plays an important role in maintaining spinal alignment and determining the degree of biomechanical loading during smartphone use. Deviations in cervical and trunk posture can increase muscle activity and mechanical strain, contributing to musculoskeletal disorders. A previous study showed that prolonged smartphone use induces significant changes in neck and trunk flexion compared with other seated activities [5]. Among smartphone gamers and eSports athletes, long periods of static sitting further increase the mechanical load on the cervical and thoracic spine, increasing the risk of developing musculoskeletal problems [6]. Prolonged cervical flexion during smartphone tasks has been linked to elevated neck muscle activity and discomfort [7, 8]. Moreover, increased neck, upper back, and shoulder pain have been associated with static postures and sustained muscle tension in handheld device users may have adverse effects on productivity and quality of life [9].

Ergonomic risk assessment tools provide an objective framework to assess postural risk during smartphone use. The Rapid Upper Limb Assessment (RULA) has been widely applied to evaluate neck, trunk, and upper‑limb postures and to estimate the risk of work‑related musculoskeletal disorders [10]. A previous study demonstrated a high ergonomic risk among smartphone users, as indicated by RULA results [11]. Variations in sitting posture, such as supported versus unsupported sitting or different leg positions, therefore have the potential to alter both spinal kinematics and RULA‑derived risk levels during smartphone tasks [12]. However, most existing research has focused on chair sitting and general smartphone tasks such as texting, browsing, and video watching, with relatively few studies examining culturally specific floor‑sitting postures during smartphone use or gaming [13]. Cultural sitting behaviors, such as cross-legged sitting on the floor, which is particularly common in Thai and other Asian populations, may further alter trunk and cervical mechanics and present different ergonomic risks compared with traditional chair-based postures. These variations in culturally influenced sitting positions warrant further investigation to understand better their impact on posture and musculoskeletal health during smartphone gaming.

Therefore, this study aimed to investigate the effects of three sitting positions, including sitting on a chair with and without a backrest, and cross-legged sitting on the floor, on cervical and trunk postures, musculoskeletal discomfort, and ergonomic risk during smartphone gaming in young adults. We hypothesize that sitting without back support or in a cross-legged posture would result in greater neck flexion, altered trunk alignment, increased musculoskeletal discomfort, and a higher ergonomic risk compared with sitting with backrest support. The findings from this study may contribute to evidence-based ergonomic recommendations and physical therapy interventions to reduce posture-related neck and back problems among habitual smartphone gamers.

## Materials and methods

### Study design

**Fig 1:**
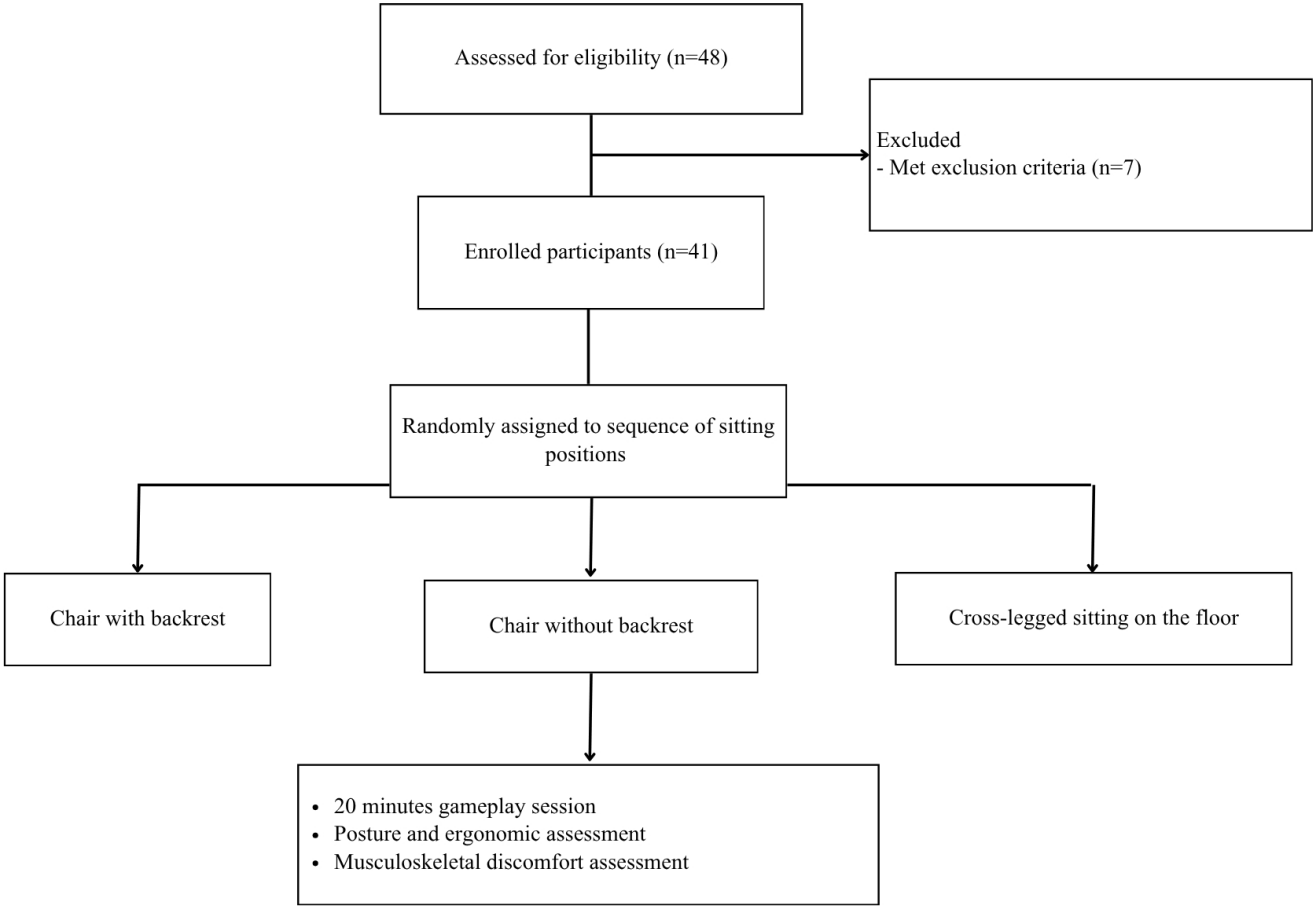
Participant flow diagram of the study.

This study employed a laboratory-based within-subject repeated-measures experimental design with randomized sequence of sitting positions. Each participant performed smartphone gaming in three different sitting conditions for 20 minutes per condition. Participant recruitment and data collection were conducted between January and July 2021 in the Physical Therapy Laboratory at Walailak University, Thailand, where temperature, lighting, and seating conditions were standardized to minimize environmental variability. The study protocol and consent procedures were reviewed and approved by the Human Research Ethics Committee at Walailak University, Thailand (Reference No. WUEC-20-222-01). All participants provided written informed consent prior to participation. All experimental procedures were conducted in accordance with the Declaration of Helsinki. This study was reported in accordance with the STROBE guidelines, where applicable. Participant flow is presented in Fig 1.

### Participants

Eligible participants were males or females aged 18–24 years who had been playing Realm of Valor (ROV) on a smartphone for at least 20 minutes per day, three days per week, for at least six months. Participants were excluded if they (1) had any musculoskeletal or neurological disorders, including the presence of any current pain at rest, a history of spinal deformity, structural abnormalities of the spine or extremities, or any diagnosed conditions affecting these regions; (2) had acute injuries or localized symptoms such as chest pain, open wounds, sharp pain, swelling, or sprains; or (3) presented with general health problems that could interfere with participation, such as fatigue, palpitations, dizziness, syncope, or uncorrected visual impairment. Participants were withdrawn from the study if they voluntarily discontinued participation before completing the protocol.

### Sample size estimation

An a priori power analysis was conducted using G*Power software (version 3.1.9.7, Germany) to determine the required sample size. The calculation was based on an F-test (ANOVA: repeated measures, within factors). A medium effect size of 0.25 was set, with a statistical power of 0.90 and an alpha level of 0.05. The number of measurements was set at five, with an assumed correlation among repeated measures of 0.3 and a nonsphericity correction (ε) of 1.0. The analysis indicated that a total of 36 participants were required. To account for an anticipated 15% dropout rate due to incomplete data, the final sample size was increased to 41 participants.

### Outcome measures

The outcomes were cervical and trunk postures (neck flexion angle and trunk angle), musculoskeletal discomfort, and ergonomic risk. All assessors underwent standardized training in accordance with the study protocol before data collection to ensure procedural consistency and measurement accuracy. The assessor responsible for cervical and trunk posture measurements was a senior physical therapist with over 20 years of clinical experience in musculoskeletal practice. Two additional assessors, who collected data on perceived musculoskeletal discomfort and ergonomic risk, were novice physical therapists with one year of relevant clinical experience; both received specific training to ensure accurate and consistent data collection.

### Cervical and trunk postures

**Fig 2:**
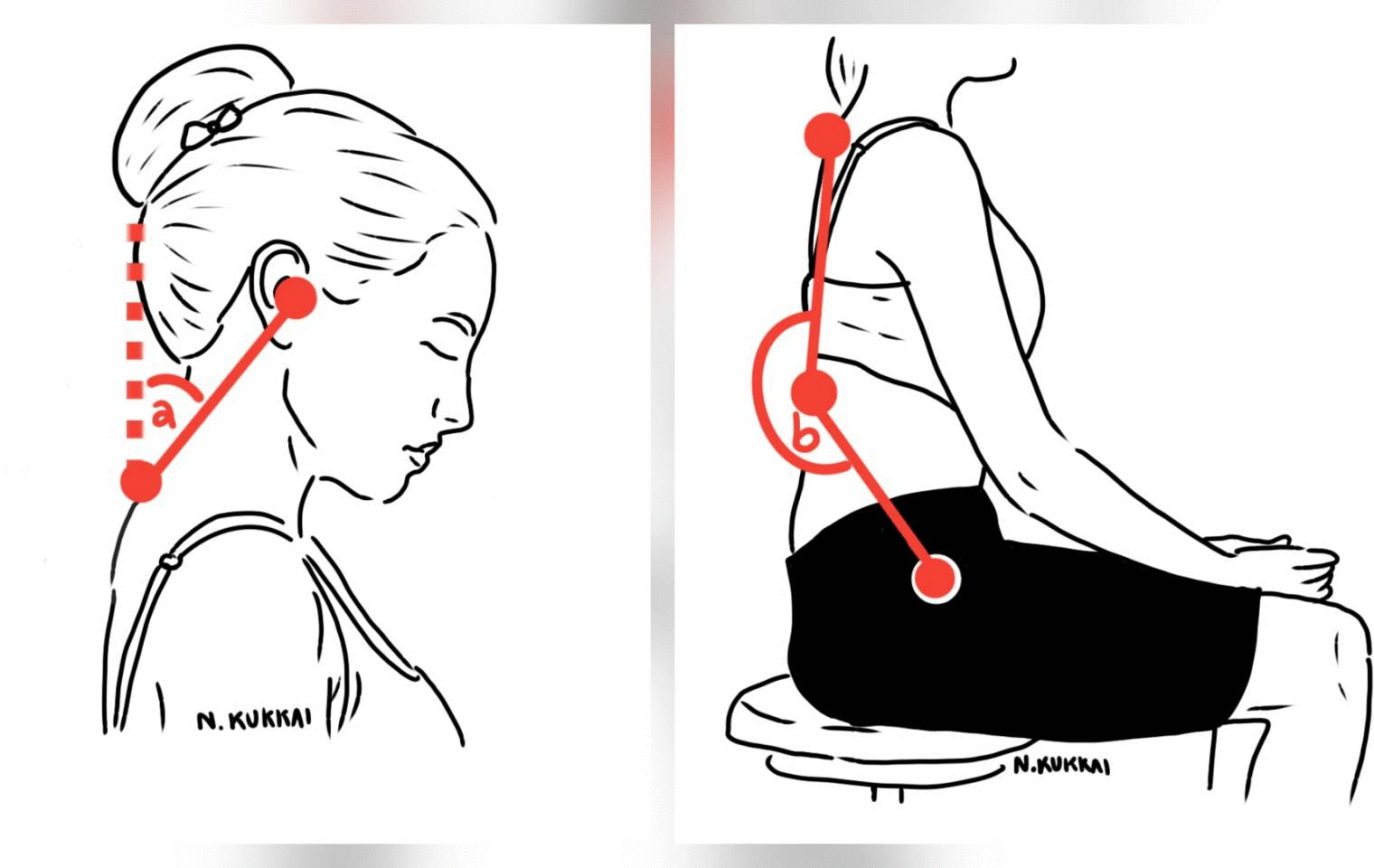
Neck flexion (a) and trunk angle (b) measurements.

The neck flexion and trunk angles were used to represent cervical and trunk postures, respectively. Anatomical markers were placed on specific landmarks, including the tragus, the spinous processes of C7 and T12, and the greater trochanter, following palpation by the same trained assessor. Participants wore fitted clothing, and openings were created at marker locations to allow direct placement on the skin, ensuring accurate identification of anatomical landmarks and avoiding marker placement over clothing. The neck flexion angle was defined as the angle formed between a line connecting the tragus and the spinous process of C7 and a vertical reference line (measured anterior to the intersection). The trunk angle was defined as the angle formed between the line connecting C7 to T12 and another connecting T12 to the greater trochanter, measured posterior to the intersection [14] (Fig 2). Digital video recordings were obtained in the sagittal plane using a high-definition camera (PAL, 25 frames per second, resolution 720 × 576 pixels; Sony HDR-PJ670, Tokyo, Japan) positioned three meters from the participant. The camera height was individually adjusted for each participant to align with the acromion process. The assessor measured and adjusted the camera so that the center of the lens was positioned at the level of the acromion process for each sitting condition. Prior to each recording, camera alignment was calibrated using a spirit level to ensure that the camera axis was parallel to the ground. Joint angle measurements were obtained from video recordings at predefined time points (0, 5, 10, 15, and 20 minutes) during each session. All angular measurements were analyzed using Kinovea™ software (version 2023.1.1), which allows two-dimensional motion analysis through frame-by-frame photographic assessment [15]. Prior to data collection, intra- and inter-rater reliability for photographic angle measurements were assessed. Two trained assessors independently measured each angle at each time point, with each measurement repeated three times. Intraclass correlation coefficients (ICC) were calculated using a two-way mixed-effects model with absolute agreement, demonstrating excellent reliability (ICC range = 0.86–0.99; corresponding 95% confidence intervals ranged from 0.444 to 0.998).

### Musculoskeletal discomfort

Musculoskeletal discomfort that occurred during the 20-minute gaming session was reported immediately after gameplay. Participants identified the location(s) using a standardized body chart, and symptom intensity was rated using a Visual Analog Scale (VAS) ranging from 0 (“no pain”) to 10 (“worst imaginable pain”) [16]. The highest intensity score reported for each location was used for analysis.

### Ergonomic risk

Ergonomic risk was evaluated using the Rapid Upper Limb Assessment (RULA), which has been used to assess ergonomic risks in smartphone users [11, 17]. RULA showed the strongest association with musculoskeletal disorder (MSD) levels and is therefore considered particularly suitable for assessing MSD risk in the upper limbs and the neck–trunk region [18]. RULA assessment was conducted continuously throughout each 20-minute session based on trained assessor observation. For analysis, the highest RULA score observed during gameplay was used to represent the maximum RULA-derived ergonomic risk for each sitting position, capturing the most adverse posture maintained during gameplay.

### Study procedure

All enrolled participants provided demographic and smartphone-related information, including age, sex, body weight, height, hand dominance, years of smartphone use, device weight (including protective case), screen size, and smartphone gaming behavior. All experimental sessions were conducted in the same laboratory room under controlled environmental conditions (temperature 25–26°C, adequate lighting, and minimal background noise).

The order of the three sitting positions, namely chair with backrest, chair without backrest, and cross-legged sitting on the floor, was randomly assigned to each participant using a lottery draw method with six predefined orders to minimize sequence effects and to help distribute condition orders across participants. The allocation sequence was concealed until assignment to prevent selection bias. A standardized armless chair with an open backrest design was used for all participants. The chair had a seat height of 47 cm, a backrest height of 41 cm (measured from the seat surface), a backrest inclination of approximately 10°, and a seat diameter of 37 cm. The no-backrest condition used a chair of the same dimensions without a backrest. If a participant’s feet did not reach the floor, a footrest was provided to maintain approximately 90 degrees of hip and knee flexion and a neutral pelvic alignment. For cross-legged sitting, participants sat on a firm yoga mat without additional cushioning. The different sitting positions used in this study are illustrated in S1 Fig. Each trial began after the participant assumed the target sitting position for at least one minute to ensure postural stabilization before recording commenced.

For each sitting condition, participants played ROV on their own smartphones to ensure natural handling and familiarity with the device. Gameplay was performed at each participant’s usual skill level, as all participants were experienced players. Each gaming session lasted 20 minutes, during which participants were instructed to maintain their posture naturally without verbal correction or interference. After completing each session, a rest period of approximately 10 minutes, or until any fatigue, muscle tightness, pain, or discomfort had resolved, was provided before proceeding to the next randomized posture, ensuring that no residual symptoms including fatigue, muscle tightness, pain, or discomfort were present prior to the subsequent condition.

During gameplay, RULA scoring was performed by a trained assessor positioned laterally to the participant, aligned with the camera location. The assessor was seated on an elevated chair to ensure a clear view of the participant’s posture and all relevant movements required for RULA evaluation. The assessor maintained a consistent observation position throughout the session but allowed limited, controlled movements within a predefined area, such as briefly standing or leaning forward, to improve visibility, when necessary, without interfering with the participant’s visual field or concentration. Standardized scoring procedures were followed to minimize potential bias. After each 20-minute session, another assessor immediately recorded the participant-reported discomfort using a standardized body chart and assessed intensity using a VAS.

### Statistical analysis

All statistical analyses were performed using SPSS for Windows version 23 (IBM Corp., Armonk, NY, USA). A p-value < 0.05 was considered statistically significant. Descriptive statistics were used to summarize participants’ demographic characteristics, gaming behavior, and smartphone usage. Continuous variables (neck flexion angle, trunk angle, symptom intensity, and RULA score) were treated as quantitative data and are presented as mean ± standard deviation (SD), while categorical variables are presented as frequencies and percentages. All statistical assumptions were verified prior to analysis, including normality (Shapiro–Wilk test). Whereas assumptions were not met, appropriate non-parametric tests were applied. No missing data was observed for any key variables.

The effects of sitting position (backrest, no-backrest, cross-legged) and time (five time points: T0–T4) on neck flexion angle and trunk angle were analyzed using a two-way repeated-measures ANOVA (3 × 5 design). The assumption of sphericity for repeated measures was assessed using Mauchly’s test. When the assumption was violated, Greenhouse–Geisser corrections were applied to adjust the degrees of freedom. When sphericity was met, results based on sphericity-assumed estimates were reported. When significant main or interaction effects were observed, Bonferroni-adjusted post-hoc pairwise comparisons were conducted. Effect sizes for the ANOVA analyses were reported as partial eta squared (η²).

Because perceived musculoskeletal discomfort intensity and ergonomic risk scores (RULA) did not meet the assumptions of normality, these variables were compared across sitting positions using the Friedman test for related samples. Post-hoc pairwise comparisons were conducted using Wilcoxon signed-rank tests with Bonferroni correction when the Friedman test indicated a significant effect. Differences in the frequency of symptom locations among sitting positions were examined using Cochran’s Q test. Kendall’s W was used to estimate effect sizes for non-parametric tests, including the Friedman test and Cochran’s Q test.

## Results

A total of 48 participants were assessed for eligibility. Of these, 7 participants met exclusion criteria. A total of 41 participants were enrolled in this study. Most participants were young adult males (85.4%) with normal body mass index and extensive experience in smartphone gaming. On average, they reported several years of smartphone use and frequent gameplay sessions. Most participants were right-hand dominant. Detailed demographic and smartphone-related characteristics are summarized in Table 1.

**Table 1:** Demographic and smartphone-related variables.

	Mean ± SD	n (%)
Sex:		
• Male		35 (85.4)
• Female		6 (14.6)
Age (years)	20.41 ± 1.13	
Weight (kg)	66.91 ± 12.31	
Height (cm)	170.91 ± 6.56	
Body Mass Index (kg/m2)	22.96 ± 3.71	
Duration of smartphone use (years)	7.70 ± 2.17	
Game playing time (mins/session)	82.92 ± 45.17	
Game playing frequency (days/week)	5.75 ± 1.28	
Device weight (including case) (g)	214.50 ± 28.40	
Screen size (inches)	5.80 ± 0.59	
Hand dominance: right		40 (97.6)

### Cervical and trunk postures

A two-way repeated measures ANOVA demonstrated a significant interaction effect between sitting position and time for neck flexion angle (Greenhouse–Geisser corrected: F(3.87, 232.27) = 5.31, p < 0.001, partial η² = 0.081), indicating that the magnitude and progression of cervical postural changes differed among sitting positions over time. Significant main effects were also found for sitting position (F(2,120) = 26.88, p < 0.001, partial η² = 0.309) and time (Greenhouse–Geisser corrected: F(1.94, 232.27) = 189.25, p < 0.001, partial η² = 0.612), suggesting temporal adaptations in cervical posture during smartphone gameplay. Post-hoc pairwise comparisons (Bonferroni-adjusted) showed that the cross-legged position demonstrated the greatest neck flexion, followed by no-backrest, whereas the backrest position showed the smallest angles. These differences were consistently observed at each time point from T1 to T4, with significantly greater neck flexion in the cross-legged position compared with both backrest and no-backrest conditions (p ≤ 0.001). Detailed pairwise comparisons for each time point are presented in S1 Table. Spaghetti plots of neck flexion for all three sitting conditions are presented in S2–S4 Figs.

**Fig 3:**
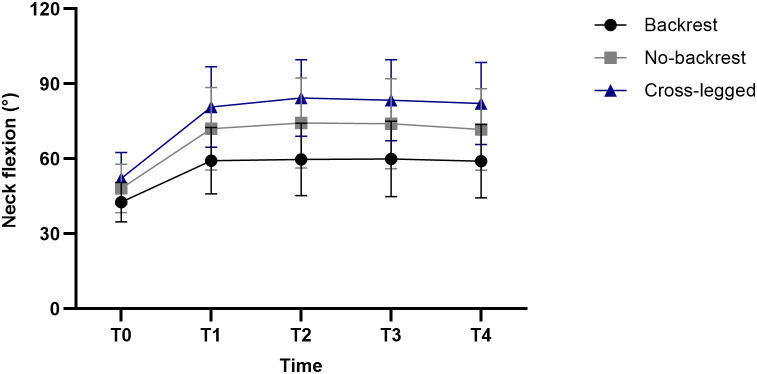
Changes in neck flexion angles over time across sitting positions during smartphone gaming.

**Fig 4:**
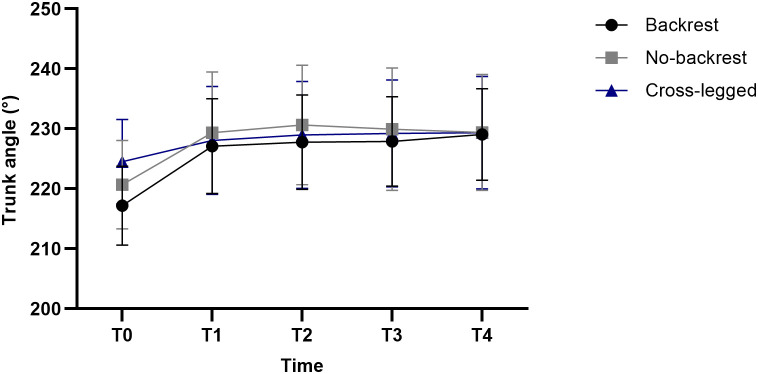
Changes in trunk angles over time across sitting positions during smartphone gaming.

For trunk posture, a significant interaction effect between sitting position and time was observed (F(8,480) = 2.73, p = 0.006, partial η² = 0.044). The main effect of sitting position was not significant (F(2,120) = 1.50, p = 0.226, partial η² = 0.024), whereas a significant main effect of time was found (F(4,480) = 43.39, p < 0.001, partial η² = 0.266). Post-hoc pairwise comparisons revealed that at baseline (T0), participants in the backrest position exhibited smaller trunk angles than those in the cross-legged position (p = 0.043), reflecting a more upright or slightly reclined trunk alignment, whereas no significant differences were observed at later time points (S2 Table). Overall, participants sitting cross-legged or without back support tended to maintain greater forward trunk inclination throughout gameplay. Spaghetti plots of trunk angle for all three sitting conditions are presented in S5–S7 Figs. The results are summarized in Table 2, and the temporal patterns of cervical and trunk postures are illustrated in Figs 3 and 4.

**Table 2:** Descriptive statistics and ANOVA results for neck flexion and trunk angle across sitting positions and time points during smartphone gaming.

Variable	Sitting Position	Mean ± SD (95% CI)					Main Effect F(df), p-value, Partial η^2^		Interaction	
		T0	T1	T2	T3	T4	Position	Time	F(df), p-value	Partial η^2^
Neck Flexion (degree)	Backrest	42.66 ± 7.93 (40.16–45.16)	59.24 ± 13.35 (55.03–63.46)	59.78 ± 14.48 (55.21–64.35)	59.93 ± 15.15 (55.15–64.71)	59.05 ± 14.66 (54.42–63.68)	F(2,120) = 26.88, p < 0.001*, partial η² = 0.309	F(1.94, 232.27) = 189.25, p < 0.001*, partial η² = 0.612	F(3.87, 232.27) = 5.31, p < 0.001*	0.081
	No-backrest	48.17 ± 9.77 (45.09–51.26)	72.05 ± 16.51 (66.84–77.26)	74.32 ± 18.08 (68.61–80.02)	74.00 ± 18.10 (68.29–79.71)	71.71 ± 16.32 (66.56–76.86)				
	Cross-legged	52.15 ± 10.43 (48.85–55.44)	80.78 ± 16.16 (75.68–85.88)	84.32 ± 15.37 (79.47–89.17)	83.44 ± 16.23 (78.32–88.56)	82.17 ± 16.38 (77.00–87.34)				
Trunk angle (degree)	Backrest	217.17 ± 6.60 (215.09–219.25)	227.12 ± 7.94 (224.61–229.63)	227.76 ± 7.88 (225.27–230.24)	227.90 ± 7.47 (225.55–230.26)	229.05 ± 7.63 (226.64–231.46)	F(2,120)= 1.50, p = 0.226, partial η² = 0.024	F(4,480) = 43.39, p < 0.001*, partial η² = 0.266	F(8,480) = 2.73, p = 0.006*	0.044
	No-backrest	220.68 ± 7.38 (218.35–223.01)	229.34 ± 10.14 (226.14–232.54)	230.63 ± 9.99 (227.48–233.79)	229.93 ± 10.21 (226.70–233.15)	229.39 ± 9.67 (226.34–232.44)				
	Cross-legged	224.54 ± 7.05 (222.31–226.76)	228.85 ± 8.90 (225.24–230.91)	228.85 ± 8.79 (226.16–231.79)	228.98 ± 9.37 (226.40–232.04)	228.93 ± 9.20 (226.38–232.30)				

Note. T0–T4 represent 0 (baseline), 5, 10, 15, and 20 minutes of sitting, respectively.

Data were analyzed using a two-way repeated-measures ANOVA. Effect sizes are reported as partial eta squared (η²).

* Significant difference at p-value<0.05.

### Musculoskeletal discomfort

All participants reported some degree of perceived musculoskeletal discomfort in the axial region (neck, shoulder girdle, upper back, and lower back) during smartphone gaming, despite the absence of resting pain at baseline. As shown in Table 3, the most frequently reported location was the neck, particularly during the cross-legged sitting condition, where approximately half of the participants (51.2%) experienced neck discomfort. The occurrence of neck discomfort differed significantly among sitting positions (p = 0.013, Kendall’s W = 0.106), while no significant differences were found for the shoulder girdle, upper back, or lower back regions (p > 0.05). In addition, the proportion of participants reporting perceived discomfort with an intensity of VAS ≥ 2 was examined to aid clinical interpretation, showing a similar pattern, with the neck remaining the most frequently affected region, particularly in the cross-legged sitting condition, while other regions showed comparable patterns across sitting positions. As presented in Table 4, symptom intensity scores did not significantly differ among sitting positions for any region (p > 0.05).

**Table 3:** Prevalence of musculoskeletal discomfort across different sitting positions during smartphone gaming.

Pain location	Backrest n (%)	No-backrest n (%)	Cross-legged n (%)	Cochran’s Q	p-value	Kendall’s W
Neck (any discomfort)	15 (36.6)	10 (24.4)	21 (51.2)	8.667	0.013*	0.106
Neck (VAS ≥ 2)	15 (36.6)	10 (24.4)	19 (46.3)		–	–
Shoulder girdle (any discomfort)	6 (14.6)	9 (22.0)	7 (17.1)	0.933	0.627	0.011
Shoulder girdle (VAS ≥ 2)	5 (12.2)	8 (19.5)	5 (12.2)		–	–
Upper back (any discomfort)	11 (26.8)	15 (36.6)	9 (22.0)	2.545	0.280	0.031
Upper back (VAS ≥ 2)	9 (22.0)	14 (34.1)	9 (22.0)		–	–
Lower back (any discomfort)	7 (17.1)	12 (29.3)	7 (17.1)	2.941	0.230	0.036
Lower back (VAS ≥ 2)	6 (14.6)	11 (26.8)	6 (14.6)		–	–

Note: Data were analyzed using Cochran’s Q test. Effect size is reported as Kendall’s W.

* Significant difference at p-value<0.05.

No statistical test was performed for VAS ≥ 2 values, as these were presented for descriptive clinical interpretation.

**Table 4:** Comparison of symptom intensity (VAS) at different body regions among sitting positions during smartphone gaming.

	Backrest		No-backrest		Cross-legged		Friedman χ²(2)	p-value	Kendall’s W
	Mean ± SD	Min – Max	Mean ± SD	Min – Max	Mean ± SD	Min – Max			
Neck	1.6 ± 2.2	0 - 6.7	1.3 ± 2.3	0 - 7.0	1.9 ± 2.1	0 - 6.5	4.717	0.095	0.058
Shoulder girdle	0.5 ± 1.3	0 - 5.5	0.9 ± 1.9	0 - 7.2	0.7 ± 1.8	0 - 6.6	0.491	0.782	0.006
Upper back	0.9 ± 1.7	0 - 5.0	1.4 ± 2.0	0 - 5.8	0.9 ± 1.8	0 - 6.1	4.375	0.112	0.053
Lower back	0.6 ± 1.4	0 - 5.2	1.0 ± 1.8	0 - 6.2	0.8 ± 2.0	0 - 7.7	2.492	0.288	0.030

Note: Data were analyzed using the Friedman test. Effect size is reported as Kendall’s W.

### Ergonomic risk

**Fig 5:**
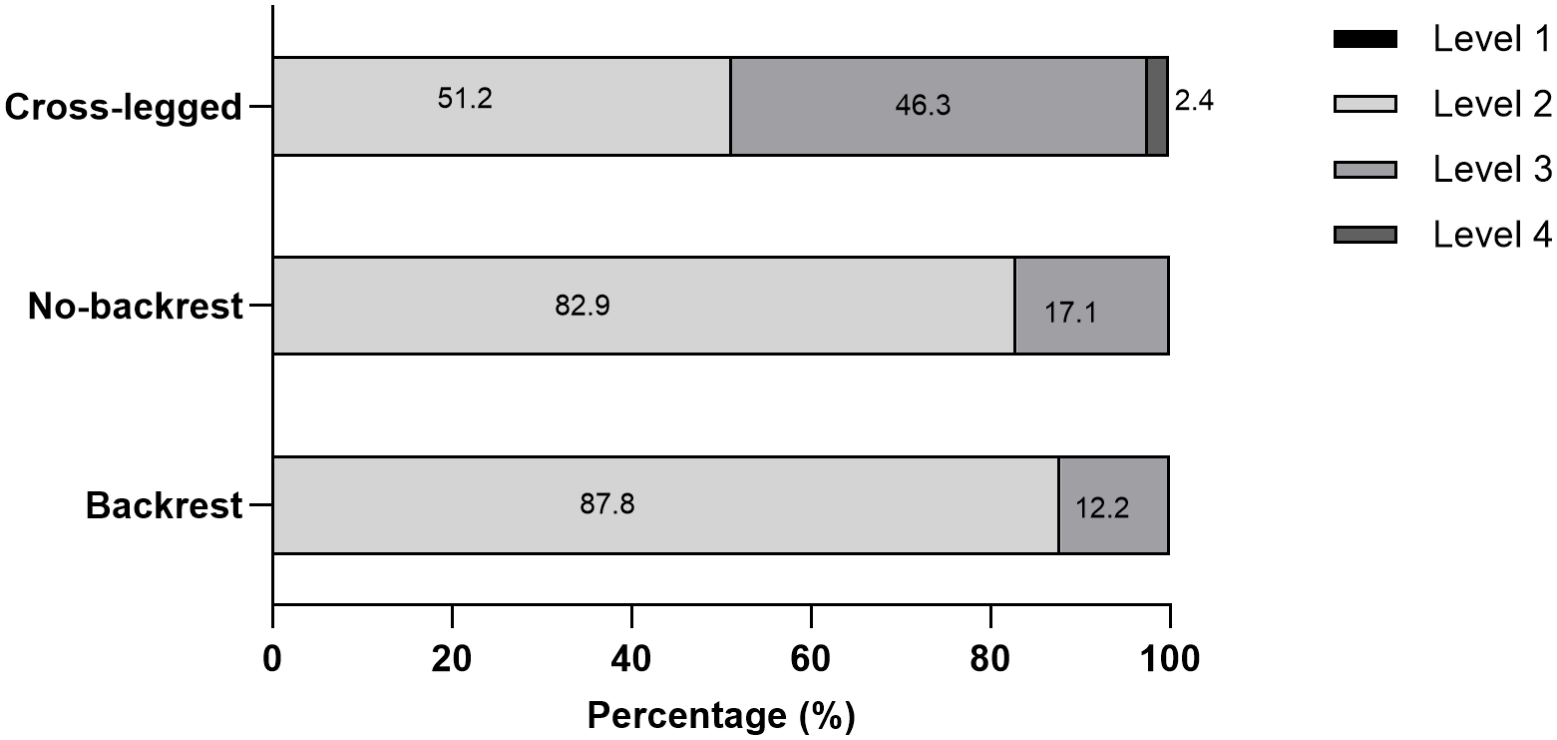
Proportion of participants at each ergonomic risk level across sitting positions.

Most participants demonstrated moderate ergonomic risk (Level 2) based on RULA assessment, as shown in Fig 5. The Friedman test revealed significant differences in both RULA scores (χ²(2) = 27.0, p < 0.001, Kendall’s W = 0.329) and RULA-based risk levels (χ²(2) = 24.88, p < 0.001, Kendall’s W = 0.303) among sitting positions. Post-hoc analysis indicated that the cross-legged sitting position had significantly higher RULA scores, and RULA-based risk levels compared with both backrest and no-backrest positions (p < 0.001). Overall, the cross-legged position was associated with the highest ergonomic risk, followed by no-backrest and backrest positions (Table 5).

**Table 5:** Comparison of RULA scores and RULA-derived ergonomic risk levels across sitting positions during smartphone gaming.

	Backrest (BR)		No-backrest (NBR)		Cross-legged (CL)		Friedman χ²(2)	p -value	Kendall’s W	Post-hoc
	Mean ± SD (95% CI)	Min – Max	Mean ± SD (95% CI)	Min – Max	Mean ± SD (95% CI)	Min – Max				
RULA score	4.2 ± 0.5 (4.0-4.3)	3.0–6.0	4.2 ± 0.6 (4.1-4.4)	4.0–6.0	4.7 ± 0.9 (4.5-5.0)	4.0–7.0	27.0	< 0.001*	0.329	CL > BR CL > NBR
RULA-based risk levels (level1–4)	2.1 ± 0.3 (2.0-2.2)	2.0–3.0	2.2 ± 0.4 (2.1-2.3)	2.0–3.0	2.5 ± 0.6 (2.3-2.7)	2.0–4.0	24.9	< 0.001*	0.303	CL > BR CL > NBR

Note: Data were analyzed using the Friedman test. Effect sizes are reported as Kendall’s W. Post hoc pairwise comparisons were conducted using Bonferroni-adjusted Wilcoxon signed-rank tests.

* Significant difference at p-value<0.05.

## Discussion

These findings showed a significant interaction between sitting position and gameplay time on both cervical and trunk postures. Sitting on a chair, either with or without a backrest, and sitting cross-legged on the floor affected posture in distinct ways. Moreover, neck discomfort and RULA-derived ergonomic risk varied significantly across positions, highlighting the direct role of posture in musculoskeletal outcomes.

Cervical and trunk postures changed gradually throughout the 20-minute gaming session compared with baseline. The significant interaction with time reflects a postural drift phenomenon, where gradual flexion develops during prolonged sitting. In addition, competitive mobile gaming, such as ROV, places high cognitive demands through sustained attention and rapid decision-making. Increased cognitive load has been linked to elevated gamma activity and may influence postural regulation mechanisms [19]. These findings are consistent with previous studies reporting increased cervical flexion during smartphone video viewing [20]. Similarly, unsupported sitting has been shown to produce greater neck flexion angles than supported sitting during smartphone use [21]. In the present study, the cross-legged sitting posture induced the greatest neck flexion, followed by sitting without a backrest, while the backrest position resulted in the smallest cervical angles. This pattern aligns with previous evidence showing poor neck posture in floor-sitting positions during smartphone use [22]. Excessive neck flexion increases activation of the upper trapezius and sternocleidomastoid muscles, contributing to cumulative strain over prolonged use [23]. Regarding trunk posture, the backrest position promoted a more neutral or slightly reclined alignment, particularly at the beginning of gameplay. However, as gaming continued, all positions demonstrated a gradual increase in trunk flexion after five minutes and remained relatively constant until the end of the session. Prolonged visual attention to smartphone screens, such as texting or watching videos, has been associated with increased trunk inclination and thoracic kyphosis compared with neutral posture [24]. However, different postural strategies for the neck and trunk during smartphone use may also depend on environmental factors, such as forearm support on a table [25].

Perceived neck discomfort was the most frequently reported symptom during smartphone gaming, especially in the cross-legged sitting position. This finding is consistent with evidence that prolonged neck flexion and static sitting increase deviation of the head and neck angle and mechanical strain on cervical structures, including the trapezius muscles, resulting in discomfort [26, 27]. However, symptom intensity did not significantly differ across sitting positions, possibly due to the relatively short exposure period. The 20-minute duration may not have been long enough to induce noticeable pain responses compared with habitual prolonged gaming over several hours [28]. A previous study has suggested that increased neck pain and decreased muscle endurance are associated with smartphone usage duration [29].

The RULA assessment revealed significant differences in RULA-derived ergonomic risk across sitting positions, with the cross-legged posture associated with the highest risk. Participants in this posture were distributed across RULA Levels 2 and 3 in nearly equal proportions, indicating moderate to high levels of RULA-based risk. These findings support a prior study showing that floor-sitting induces abnormal postural alignment, which increases ergonomic risk scores [22]. However, most participants in the chair-sitting conditions demonstrated moderate RULA-based risk (Level 2), consistent with previous research [30]. Another study identified Level 3 risk in smartphone users, which may be explained by differences in task type and usage conditions, as previous studies involved texting and browsing under natural use without time constraints and without standardized control of posture or support conditions [11]. Moreover, a previous study has shown that even 10 minutes of smartphone texting can induce postures associated with elevated ergonomic risk [31]. This moderate to high level of RULA-based risk suggests that postural conditions may be suboptimal for sustained activity and may benefit from ergonomic modification or posture training to reduce musculoskeletal strain. Consequently, interventions promoting supported sitting and adjustable seating may help improve postural alignment and reduce perceived musculoskeletal risk during smartphone gaming.

From an ergonomic perspective, these findings emphasize the importance of sitting posture in influencing cervical and trunk alignment and perceived musculoskeletal discomfort during smartphone use. Physical therapists and ergonomists may promote posture awareness by encouraging users to maintain an upright trunk and avoid sustained neck flexion to improve postural alignment. Implementing posture-based interventions, such as active breaks or cervical stabilization exercises, may help support neck function and reduce perceived musculoskeletal strain during smartphone gaming.

This study has some limitations. Most participants were young adult males, which limits generalizability to females and older adults who may differ in musculoskeletal vulnerability and symptom patterns. The inclusion of cross-legged sitting, a culturally influenced posture, may limit generalizability to populations with different sitting habits. The 20-minute gaming duration may not fully represent real-world conditions where gaming sessions often extend beyond one hour. Although randomization and rest periods were implemented, residual effects related to cognitive load or postural fatigue cannot be entirely excluded. In addition, RULA assessment was primarily conducted from a lateral view to minimize interference with participant concentration, which may have limited the ability to capture some postural deviations outside the lateral view, and the use of the maximum score may have overestimated ergonomic risk. Furthermore, allowing participants to use their own smartphones and play at their usual skill level may have introduced variability in viewing distance, posture, and cognitive load. Although 2D photographic assessment demonstrated high reliability and is practical for clinical use, it may not capture postural changes outside the plane of observation and is less accurate than 3D motion analysis. Therefore, the findings should be interpreted with caution. Future studies should include more diverse populations and consider a wider range of sitting behaviors and usage contexts to improve generalizability. In addition, standardizing device characteristics or incorporating gameplay-related variables as covariates may help reduce variability. Intervention studies examining ergonomic aids or posture training programs may further clarify strategies to reduce musculoskeletal risks during smartphone gaming.

## Conclusions

Different sitting positions during smartphone gaming resulted in distinct cervical and trunk postures and varying ergonomic risks. The cross-legged posture produced greater cervical and trunk flexion, a higher prevalence of neck discomfort, and a greater ergonomic risk than supported sitting. The emergence of musculoskeletal discomfort suggests the presence of early musculoskeletal problems associated with sustained flexed postures. The results provide a basis for developing ergonomic recommendations and targeted interventions to reduce postural strain during smartphone use among young adults and may help support musculoskeletal health and well-being, although longitudinal studies are needed to confirm long-term effects.

## Supporting information

S1 Table Pairwise comparison of neck flexion. (DOCX)

S2 Table Pairwise comparison of trunk angle. (DOCX)

S1 Fig The three sitting positions: chair with backrest (A), chair without backrest (B), and cross-legged sitting (C). The volunteer model provided written informed consent for publication. Facial features were obscured to maintain anonymity.
(TIF)

S2 Fig Spaghetti plot of neck flexion across time in the backrest sitting condition. Thin gray lines represent individual participants, and the solid blue line indicates the mean with 95% confidence intervals. Time points T0–T4 correspond to 0, 5, 10, 15, and 20 minutes of smartphone gaming, respectively.
(TIF)

S3 Fig Spaghetti plot of neck flexion across time in the no-backrest sitting condition. Thin gray lines represent individual participants, and the solid blue line indicates the mean with 95% confidence intervals. Time points T0–T4 correspond to 0, 5, 10, 15, and 20 minutes of smartphone gaming, respectively.
(TIF)

S4 Fig Spaghetti plot of neck flexion across time in the cross-legged sitting condition. Thin gray lines represent individual participants, and the solid blue line indicates the mean with 95% confidence intervals. Time points T0–T4 correspond to 0, 5, 10, 15, and 20 minutes of smartphone gaming, respectively.
(TIF)

S5 Fig Spaghetti plot of trunk angle across time in the backrest sitting condition. Thin gray lines represent individual participants, and the solid blue line indicates the mean with 95% confidence intervals. Time points T0–T4 correspond to 0, 5, 10, 15, and 20 minutes of smartphone gaming, respectively.
(TIF)

S6 Fig Spaghetti plot of trunk angle across time in the no-backrest sitting condition. Thin gray lines represent individual participants, and the solid blue line indicates the mean with 95% confidence intervals. Time points T0–T4 correspond to 0, 5, 10, 15, and 20 minutes of smartphone gaming, respectively.
(TIF)

S7 Fig Spaghetti plot of trunk angle across time in the cross-legged sitting condition. Thin gray lines represent individual participants, and the solid blue line indicates the mean with 95% confidence intervals. Time points T0–T4 correspond to 0, 5, 10, 15, and 20 minutes of smartphone gaming, respectively.
(TIF)
