# Supplementary material for: Immediate effects of sitting position on cervical and trunk posture, perceived discomfort, and RULA-based ergonomic risk during smartphone gaming: A randomized within-subject study
Source: PLoS One. 2026 Jul 21;21(7):e0354360. doi: 10.1371/journal.pone.0354360 (PMC13387541; doi:10.1371/journal.pone.0354360)
Supplement: S1 Table — (DOCX) [file pone.0354360.s001.docx]

**S1.Table Within- and between-group pairwise comparisons of neck flexion angle across time points (T0–T4)**

| **Within group** | **T0-T1** | **T0-T2** | **T0-T3** | **T0-T4** | **T1-T2** | **T1-T3** | **T1-T4** | **T2-T3** | **T2-T4** | **T3-T4** |
| --- | --- | --- | --- | --- | --- | --- | --- | --- | --- | --- |
|  | **Mean difference (95% CI), p-value** | | | | | | | | | |
| BR | -16.585 (-23.567, -9.604),  < 0.001 | -17.122 (-24.690, -9.554),  < 0.001 | -17.268 (-25.077, -9.460),  < 0.001 | -16.390 (-24.122, -8.659),  < 0.001 | -0.537 (-3.492, 2.419), 1.000 | -0.683 (-4.080, 2.714), 1.000 | 0.195 (-3.719, 4.109), 1.000 | -0.146 (-3.220, 2.928), 1.000 | 0.732 (-3.317, 4.781), 1.000 | 0.878 (-2.932, 4.688), 1.000 |
| NBR | -23.878 (-30.859, -16.897), < 0.001 | -26.146 (-33.714, -18.579),  < 0.001 | -25.829 (-33.638, -18.021),  < 0.001 | -23.537 (-31.268, -15.805),  < 0.001 | -2.268 (-5.224, 0.687), 0.301 | -1.951 (-5.348, 1.446), 1.000 | 0.341 (-3.573, 4.255), 1.000 | 0.317 (-2.757, 3.391), 1.000 | 2.610 (-1.439, 6.659), 0.679 | 2.293 (-1.517, 6.102), 0.878 |
| CL | -28.634 (-35.615, -21.653),  < 0.001 | -32.171 (-39.738, -24.603),  < 0.001 | -31.293 (-39.101, -23.484),  < 0.001 | -30.024 (-37.756, -22.293),  < 0.001 | -3.537 (-6.492, -0.581), 0.009 | -2.659 (-6.056, 0.739), 0.268 | -1.390 (-5.304, 2.524), 1.000 | 0.878 (-2.196, 3.952), 1.000 | 2.146 (-1.903, 6.196), 1.000 | 1.268 (-2.541, 5.078), 1.000 |
| **Between group** | **T0** | | **T1** | | **T2** | | **T3** | | **T4** | |
|  | **Mean difference (95% CI)** | **p-value** | **Mean difference (95% CI)** | **p-value** | **Mean difference (95% CI)** | **p-value** | **Mean difference (95% CI)** | **p-value** | **Mean difference (95% CI)** | **p-value** |
| BR vs.NBR | -5.512 (-10.573, -0.451) | 0.028 | -12.805 (-21.066, -4.544) | 0.001 | -14.537 (-23.142, -5.931) | < 0.001 | -14.073 (-22.942, -5.205) | 0.001 | -12.659 (-21.135, -4.182) | 0.001 |
| BR vs.CL | -9.488 (-14.549, -4.427) | < 0.001 | -21.537 (-29.797, -13.276) | < 0.001 | -24.537 (-33.142, -15.931) | < 0.001 | -23.512 (-32.381, -14.644) | < 0.001 | -23.122 (-31.598, -14.646) | < 0.001 |
| NBR vs. CL | -3.976 (-9.037, 1.085) | 0.177 | -8.732 (-16.993, -0.471) | 0.035 | -10.000 (-18.606, -1.394) | 0.017 | -9.439 (-18.307, -0.571) | 0.033 | -10.463 (-18.940, -1.987) | 0.010 |

Note. T0–T4 represent 0 (baseline), 5, 10, 15, and 20 minutes of sitting, respectively.

BR=Backrest chair sitting, NBR = No-backrest chair sitting, CL=cross-legged sitting

p-values are Bonferroni-adjusted; significance was set at p < 0.05.
