# Supplementary material for: Immediate effects of sitting position on cervical and trunk posture, perceived discomfort, and RULA-based ergonomic risk during smartphone gaming: A randomized within-subject study
Source: PLoS One. 2026 Jul 21;21(7):e0354360. doi: 10.1371/journal.pone.0354360 (PMC13387541; doi:10.1371/journal.pone.0354360)
Supplement: S2 Table — (DOCX) [file pone.0354360.s002.docx]

**S2.Table Within- and between-group pairwise comparisons of trunk angle across time points (T0–T4)**

| **Within group** | **T0-T1** | **T0-T2** | **T0-T3** | **T0-T4** | **T1-T2** | **T1-T3** | **T1-T4** | **T2-T3** | **T2-T4** | **T3-T4** |
| --- | --- | --- | --- | --- | --- | --- | --- | --- | --- | --- |
|  | **Mean difference (95% CI), p-value** | | | | | | | | | |
| BR | -9.951 (-13.883, -6.020),  < 0.001 | -10.585 (-14.655, -6.515),  < 0.001 | -10.732 (-14.793, -6.670),  < 0.001 | -11.878 (-15.815, -7.941),  < 0.001 | -0.634 (-4.411, 3.143), 1.000 | -0.780 (-4.329, 2.768), 1.000 | -1.927 (-6.018, 2.165), 1.000 | -0.146 (-3.868, 3.575), 1.000 | -1.293 (-5.025, 2.440), 1.000 | -1.146 (-4.855, 2.562), 1.000 |
| NBR | -8.659 (-12.590, -4.727),  < 0.001 | -9.951 (-14.021, -5.881),  < 0.001 | -9.244 (-13.305, -5.183),  < 0.001 | -8.707 (-12.644, -4.770),  < 0.001 | -1.293 (-5.069, 2.484), 1.000 | -0.585 (-4.134, 2.963), 1.000 | -0.049 (-4.140, 4.043), 1.000 | 0.707 (-3.014, 4.429), 1.000 | 1.244 (-2.489, 4.976), 1.000 | 0.537 (-3.172, 4.245), 1.000 |
| CL | -4.317 (-8.249, -0.386), 0.021 | -4.317 (-8.387, -0.247), 0.030 | -4.439 (-8.500, -0.378), 0.022 | -4.390 (-8.327, -0.453), 0.018 | 0.000 (-3.777, 3.777), 1.000 | -0.122 (-3.671, 3.427), 1.000 | -0.073 (-4.165, 4.018), 1.000 | -0.122 (-3.844, 3.600), 1.000 | -0.073 (-3.806, 3.659), 1.000 | -0.049 (-3.757, 3.660), 1.000 |
| **Between group** | **T0** | | **T1** | | **T2** | | **T3** | | **T4** | |
|  | **Mean difference (95% CI)** | **p-value** | **Mean difference (95% CI)** | **p-value** | **Mean difference (95% CI)** | **p-value** | **Mean difference (95% CI)** | **p-value** | **Mean difference (95% CI)** | **p-value** |
| BR vs.NBR | -3.512 (-7.275, 0.250) | 0.076 | -2.220 (-7.067, 2.628) | 0.806 | -2.878 (-7.665, 1.908) | 0.441 | -2.024 (-6.898, 2.850) | 0.946 | -0.341 (-5.100, 4.417) | 1.000 |
| BR vs.CL | -7.366 (-11.128, -3.603) | < 0.001 | -1.732 (-6.579, 3.116) | 1.000 | -1.098 (-5.884, 3.689) | 1.000 | -1.073 (-5.947, 3.801) | 1.000 | 0.122 (-4.636, 4.880) | 1.000 |
| NBR vs. CL | -3.854 (-7.616, -0.091) | 0.043 | 0.488 (-4.360, 5.336) | 1.000 | 1.780 (-3.006, 6.567) | 1.000 | 0.951 (-3.923, 5.825) | 1.000 | 0.463 (-4.295, 5.222) | 1.000 |

Note. T0–T4 represent 0 (baseline), 5, 10, 15, and 20 minutes of sitting, respectively.

BR=Backrest chair sitting, NBR = No-backrest chair sitting, CL=cross-legged sitting

p-values are Bonferroni-adjusted; significance was set at p < 0.05.
