# Supplementary figures and images for: Immediate effects of sitting position on cervical and trunk posture, perceived discomfort, and RULA-based ergonomic risk during smartphone gaming: A randomized within-subject study
Source: PLoS One. 2026 Jul 21;21(7):e0354360. doi: 10.1371/journal.pone.0354360 (PMC13387541; doi:10.1371/journal.pone.0354360)

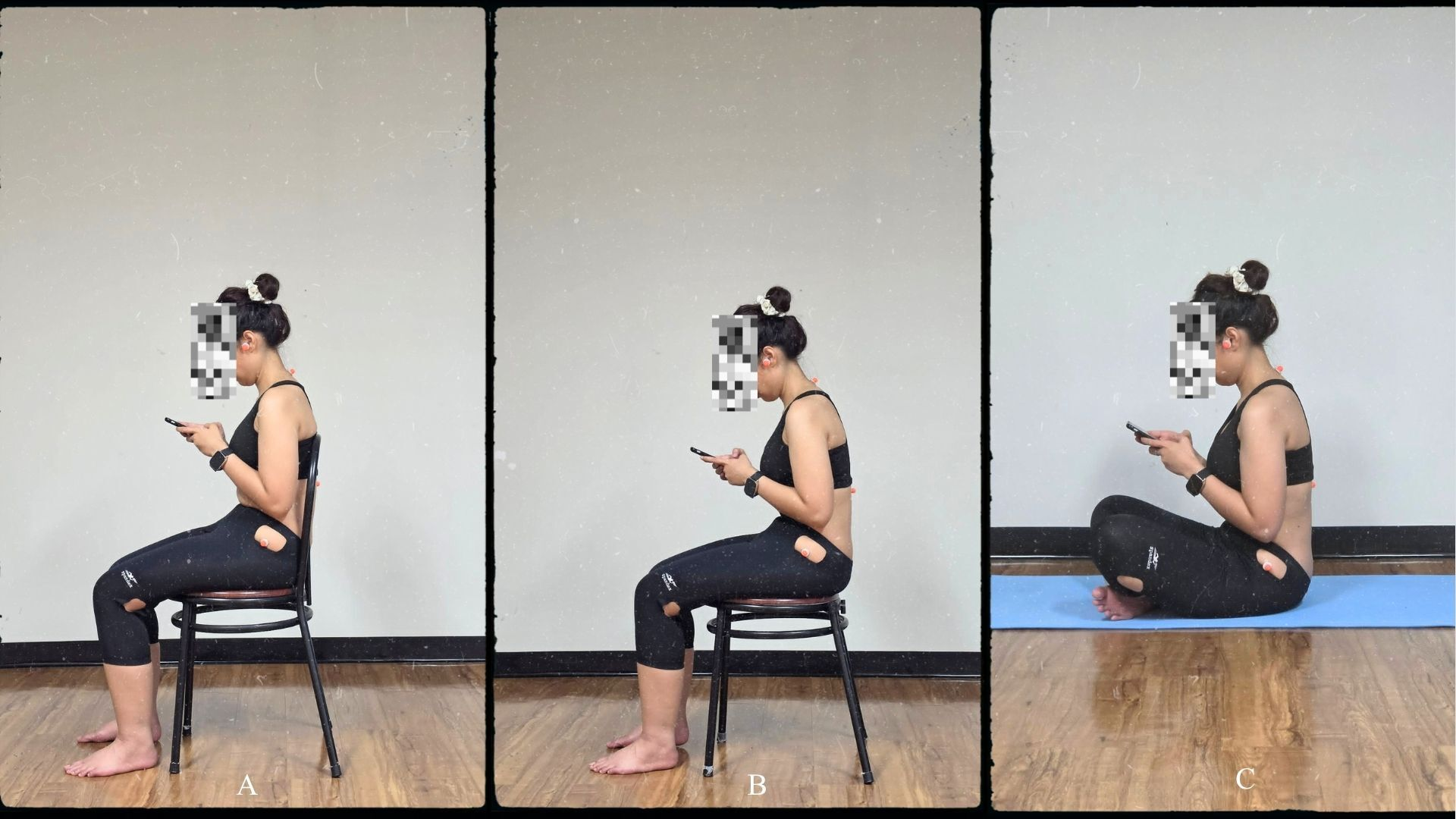

Supplement: S1 Fig — The volunteer model provided written informed consent for publication. Facial features were obscured to maintain anonymity. (TIF) [file pone.0354360.s003.tif]

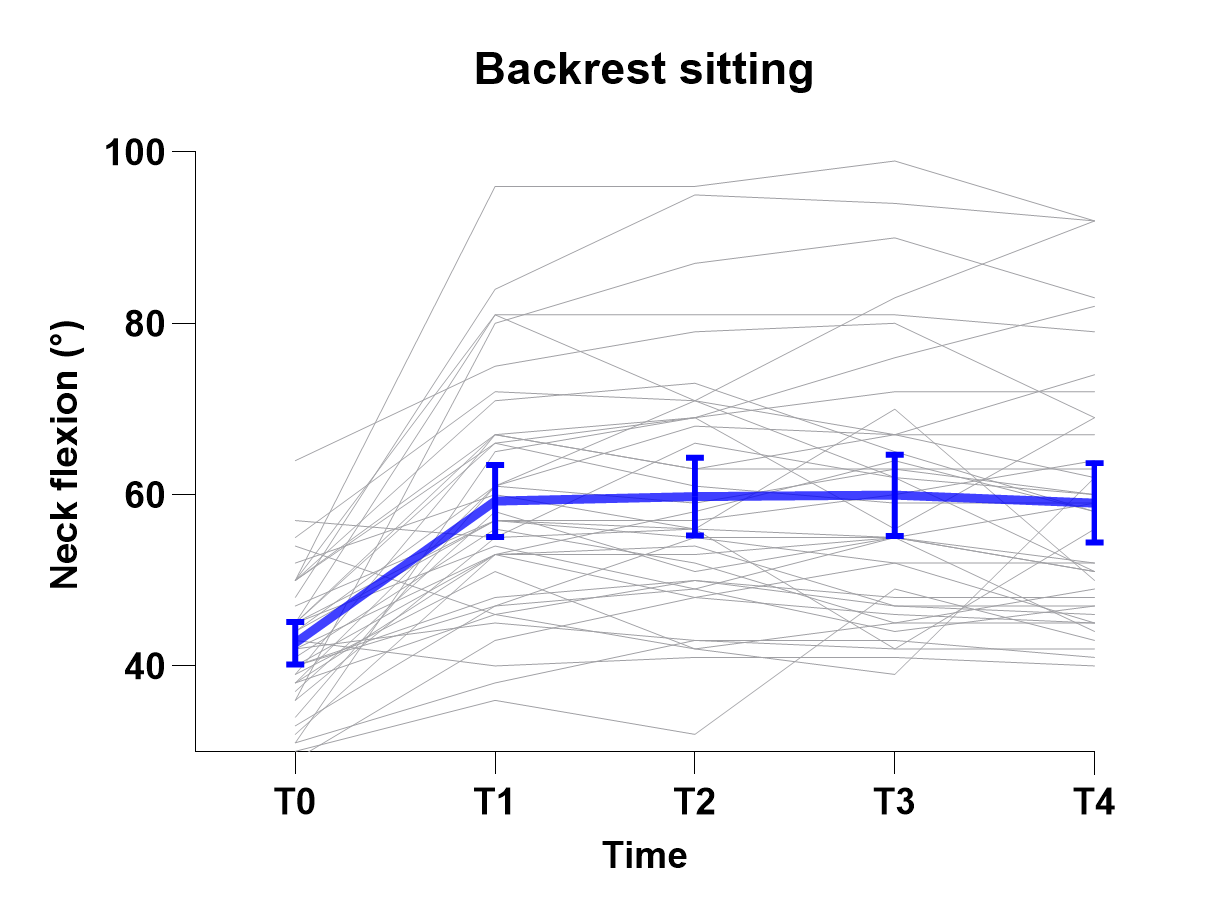

Supplement: S2 Fig — Thin gray lines represent individual participants, and the solid blue line indicates the mean with 95% confidence intervals. Time points T0–T4 correspond to 0, 5, 10, 15, and 20 minutes of smartphone gaming, respectively. (TIF) [file pone.0354360.s004.tif]

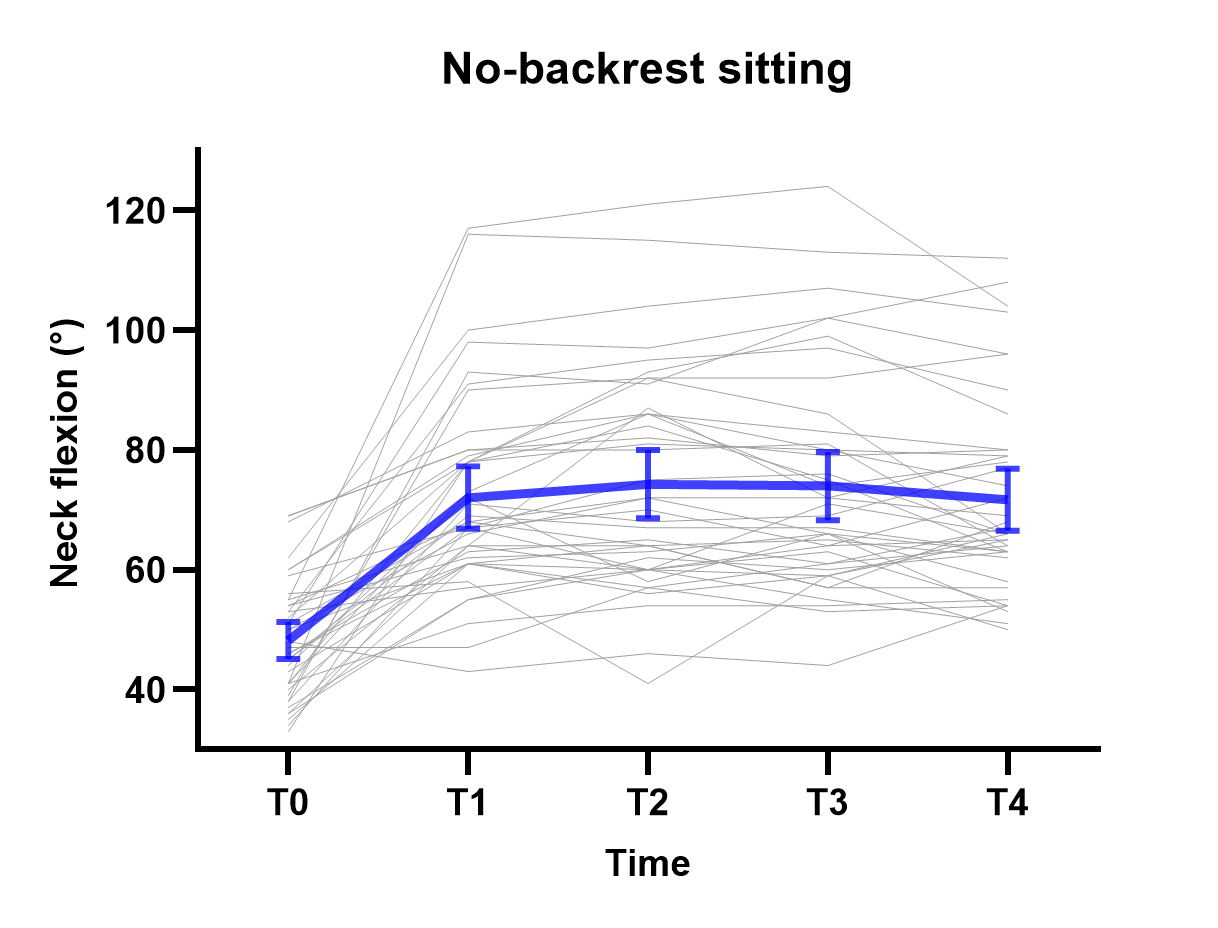

Supplement: S3 Fig — Thin gray lines represent individual participants, and the solid blue line indicates the mean with 95% confidence intervals. Time points T0–T4 correspond to 0, 5, 10, 15, and 20 minutes of smartphone gaming, respectively. (TIF) [file pone.0354360.s005.tif]

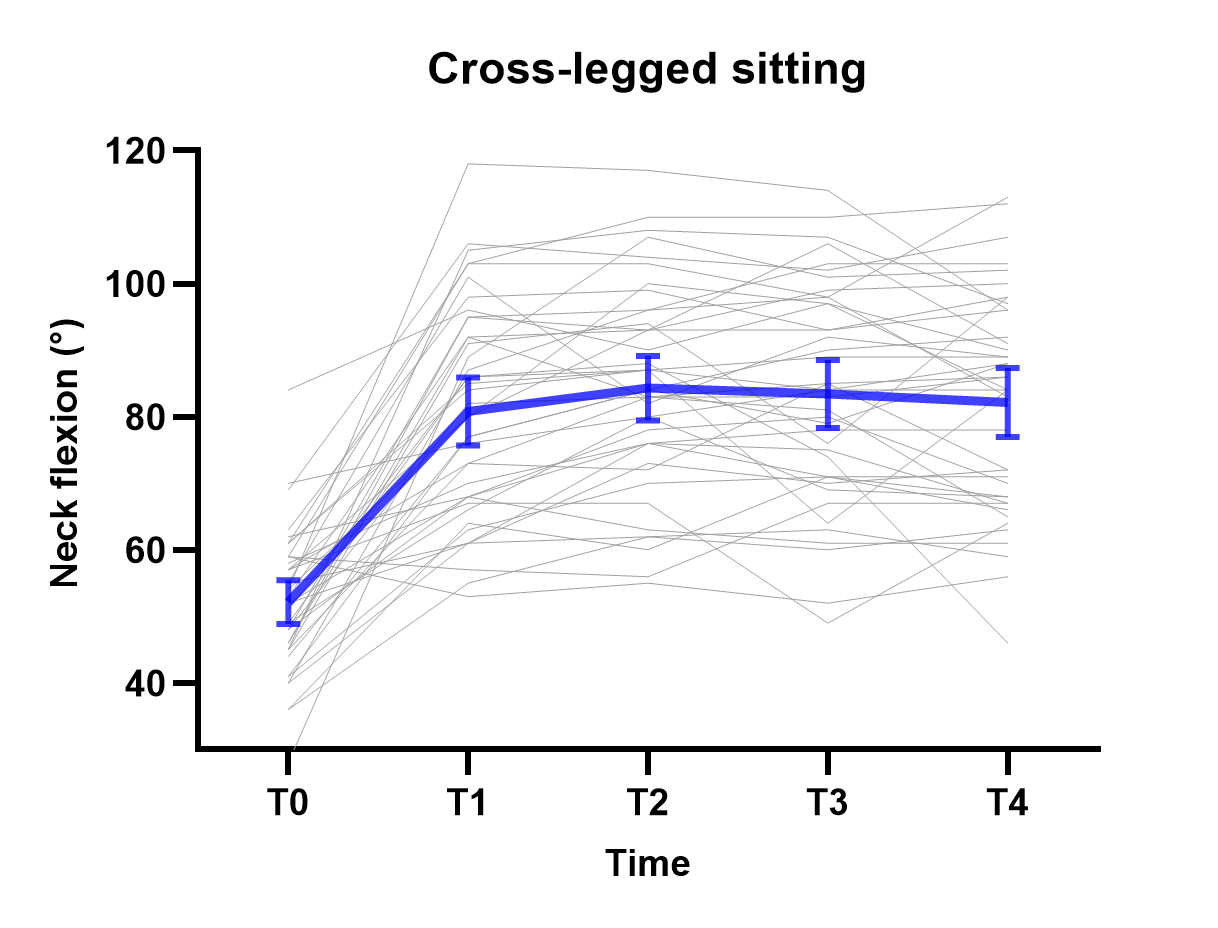

Supplement: S4 Fig — Thin gray lines represent individual participants, and the solid blue line indicates the mean with 95% confidence intervals. Time points T0–T4 correspond to 0, 5, 10, 15, and 20 minutes of smartphone gaming, respectively. (TIF) [file pone.0354360.s006.tif]

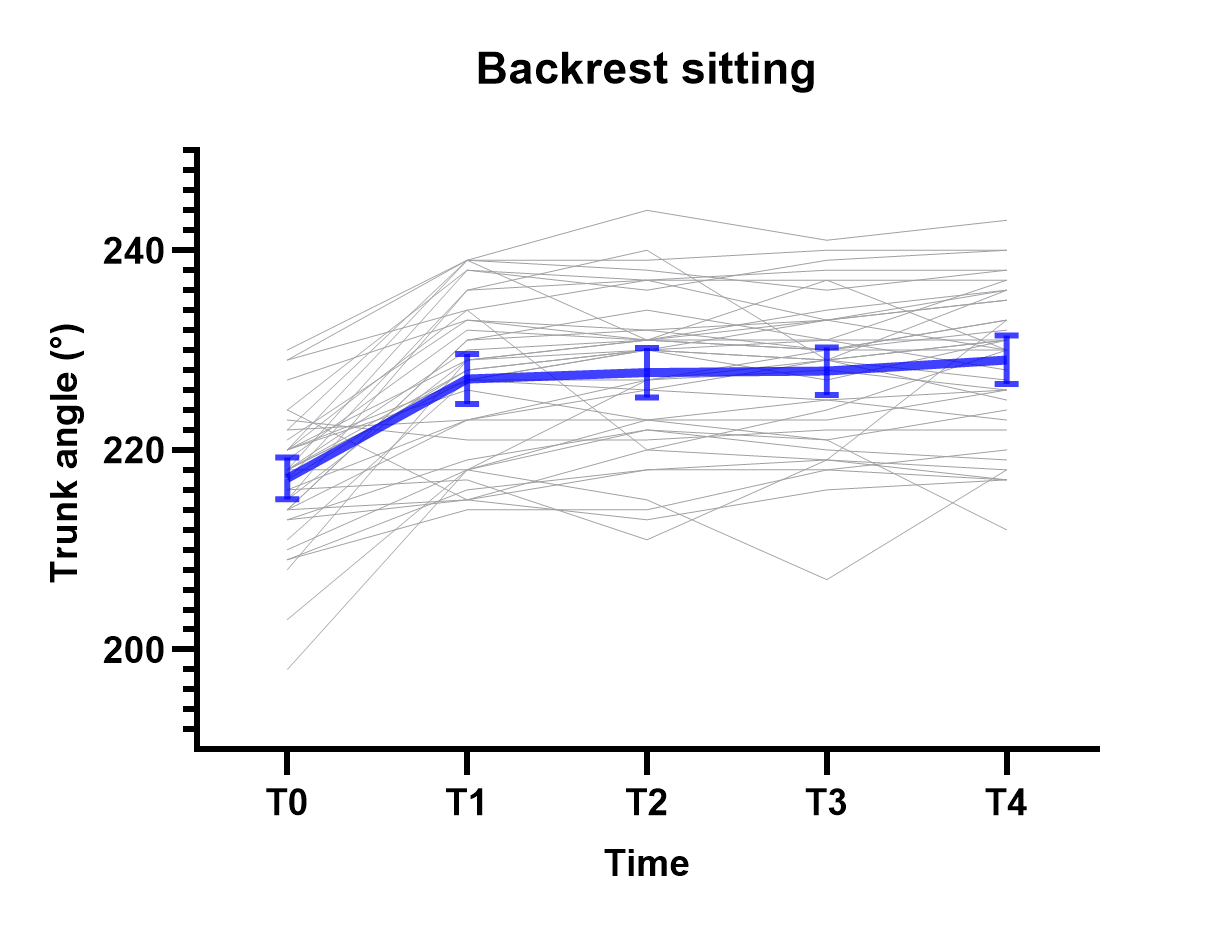

Supplement: S5 Fig — Thin gray lines represent individual participants, and the solid blue line indicates the mean with 95% confidence intervals. Time points T0–T4 correspond to 0, 5, 10, 15, and 20 minutes of smartphone gaming, respectively. (TIF) [file pone.0354360.s007.tif]

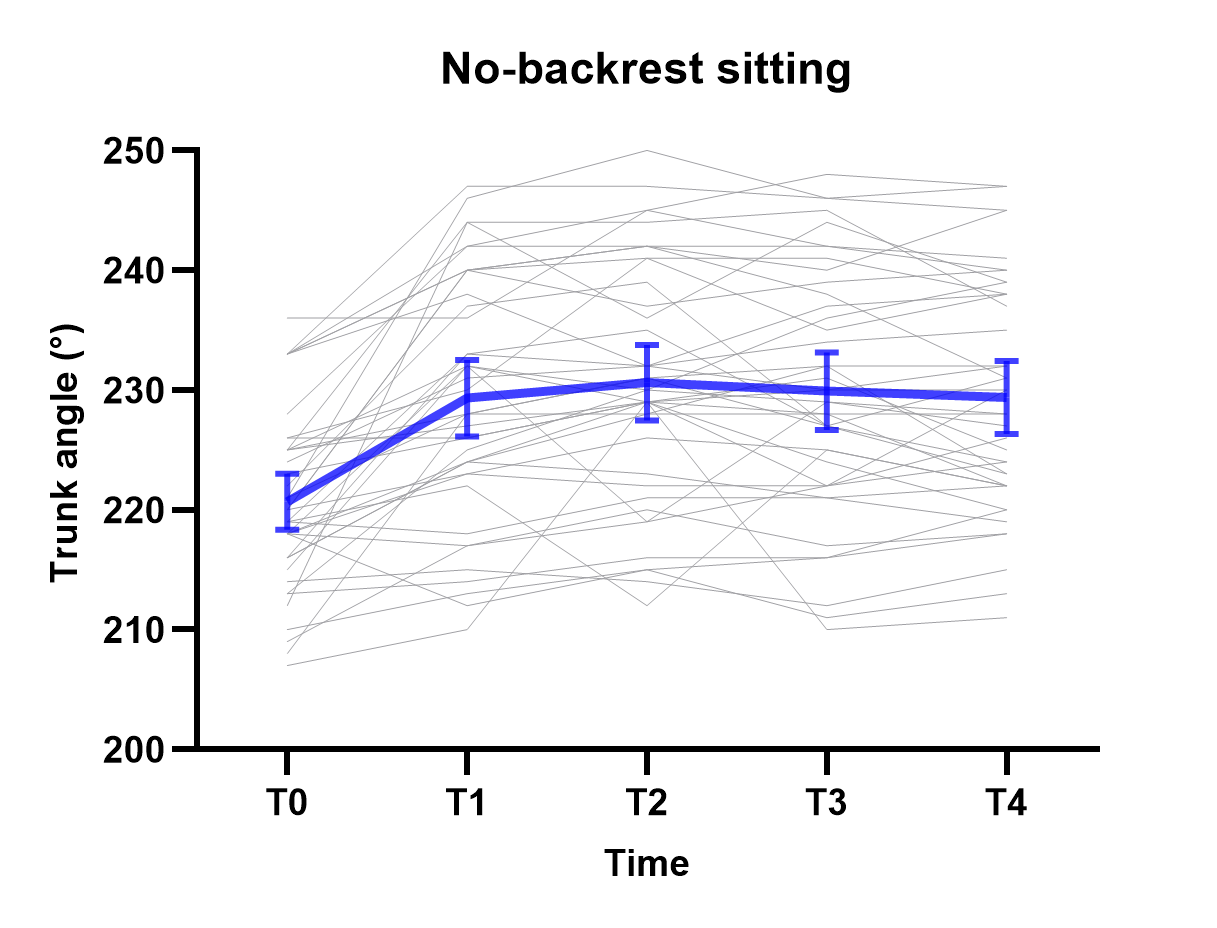

Supplement: S6 Fig — Thin gray lines represent individual participants, and the solid blue line indicates the mean with 95% confidence intervals. Time points T0–T4 correspond to 0, 5, 10, 15, and 20 minutes of smartphone gaming, respectively. (TIF) [file pone.0354360.s008.tif]

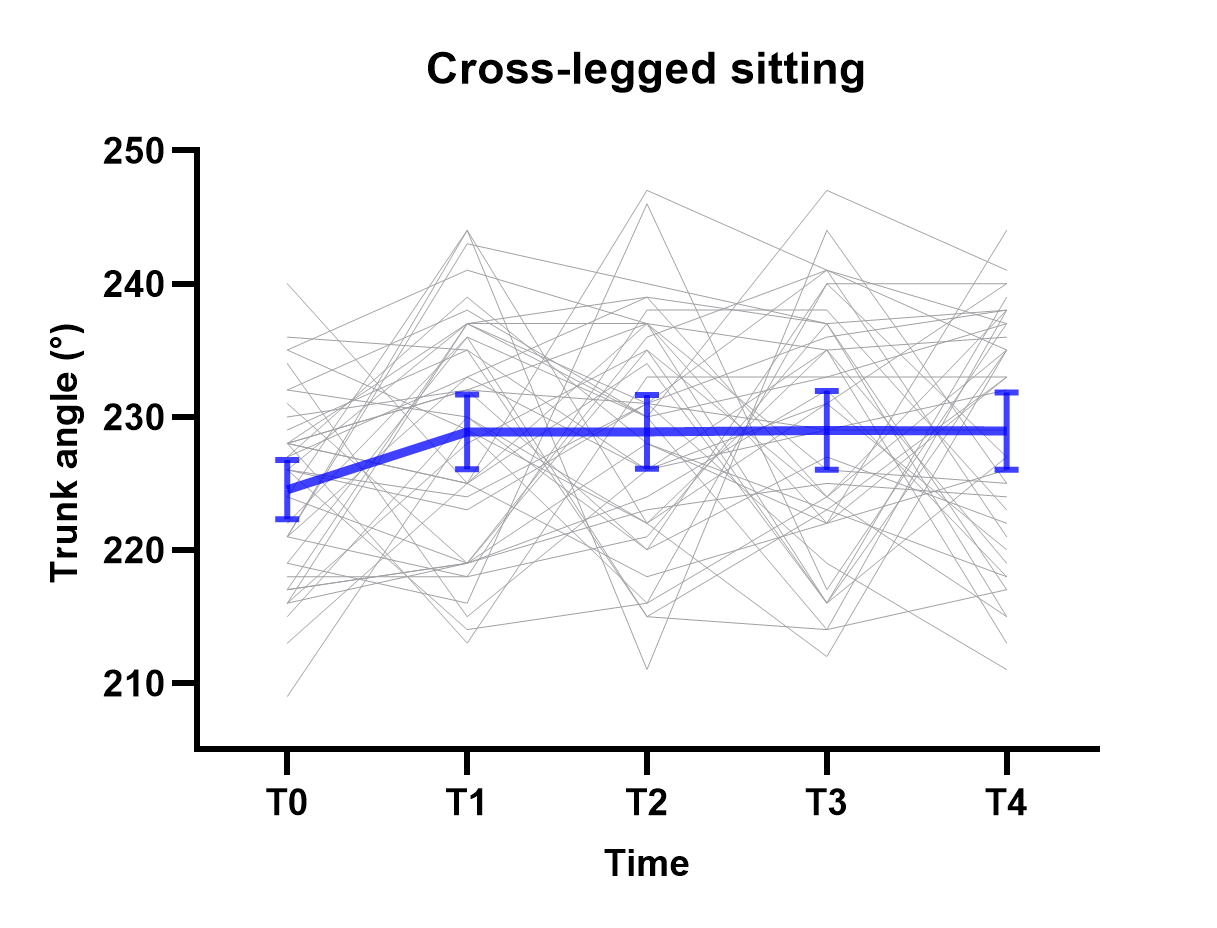

Supplement: S7 Fig — Thin gray lines represent individual participants, and the solid blue line indicates the mean with 95% confidence intervals. Time points T0–T4 correspond to 0, 5, 10, 15, and 20 minutes of smartphone gaming, respectively. (TIF) [file pone.0354360.s009.tif]
