# Supplementary material for: Immediate effects of sitting position on cervical and trunk posture, perceived discomfort, and RULA-based ergonomic risk during smartphone gaming: A randomized within-subject study
Source: PLoS One. 2026 Jul 21;21(7):e0354360. doi: 10.1371/journal.pone.0354360 (PMC13387541; doi:10.1371/journal.pone.0354360)
Supplement: Attachment — Submitted filename: Review.pdf [file pone.0354360.s010.pdf]

Review Manuscript entitled "Effect of Sitting Position on Cervical and Trunk Posture, Musculoskeletal Pain, and Ergonomic Risk During Smartphone Gaming" (PONE-D-26-01989)

Dr. Mshari Alghadier
Academic Editor
PLOS One

Dear Authors

I enjoyed reading your paper. The study concerns an important problem of effect of sitting position on cervical and trunk posture, musculoskeletal pain, and ergonomic risk during smartphone gaming. The study aimed to analyze the effects of three sitting positions, including chair sitting with and without a backrest and cross-legged sitting on the floor, on cervical and trunk postures, musculoskeletal pain or discomfort, and ergonomic risk during smartphone gaming among young adults.

However, I have a couple of minor requests to be revised as stated below.

Comments:

1. Lines 145-158: I propose adding figures illustrating the assessed cervical and trunk parameters.
2. Line 173: "*All assesors…*" Who were the assesors and what was their experience?
3. Lines 222-223: *"(...), 7 participants did not meet the inclusion and exclusion criteria"*. I don't understand the phrasing that the participants did not meet the exclusion criteria.
4. Table 1: Where can I find the data related to the female participants?
5. I suggest adding photos of the sitting positions.
