# Supplementary material for: Immediate effects of sitting position on cervical and trunk posture, perceived discomfort, and RULA-based ergonomic risk during smartphone gaming: A randomized within-subject study
Source: PLoS One. 2026 Jul 21;21(7):e0354360. doi: 10.1371/journal.pone.0354360 (PMC13387541; doi:10.1371/journal.pone.0354360)
Supplement: Attachment — Submitted filename: Response to reviewer.pdf [file pone.0354360.s012.pdf]

Response to reviewer

Reviewer 1

Major comments (substantive issues to address)

Reviewer's comment

1. Statistical reporting and analysis

o Please can you check if your repeated measures ANOVA degrees of freedom align with N=41?

Author response

Thank you for this important comment. We acknowledge that the degrees of freedom reported in the original manuscript were derived from an incorrect SPSS output table, which led to inconsistencies in the statistical reporting. Importantly, this issue was limited to the reporting of results, and the underlying analyses and overall findings remain unchanged. We have carefully reviewed all statistical outputs and have revised the manuscript to ensure that the reported F-values, degrees of freedom, and associated statistics are accurately presented based on the appropriate within- and between-subjects effects tables. In addition, we have expanded the Statistical Analysis section to clarify the analytical approach, including the handling of sphericity assumptions and the application of Greenhouse–Geisser corrections where appropriate. These revisions aim to improve transparency and provide clearer justification for the reported test statistics.

Revision made Page 11/ Line 243-246
Page 13/ Line 273-277
Page 14/ Line 287-289
Page 15/ Table 2

Reviewer's comment

2. RULA scoring validity from sagittal only assessment

o RULA requires inputs that can involve non sagittal planes (e.g., shoulder abduction, wrist deviation). Scoring from sagittal view alone can misclassify risk.

o You also use the maximum observed RULA as the representative score; this may overstate risk relative to typical postures.

o You also use the maximum observed RULA as the representative score; this may overstate risk relative to typical postures.

o Actions: Clearly state how full RULA items were operationalized from a single view; acknowledge limitations; temper claims to "RULA derived risk from sagittal observation"; consider also reporting a typical/mid session or median RULA score in the Results (or a sensitivity analysis) alongside the maximum.

Author response

Thank you for these valuable comments. First, we acknowledge that the original wording "sagittal plane observation" may have been misleading. In practice, RULA scoring was conducted through direct observation by a trained assessor positioned laterally within an area that allowed adequate

visualization of the postural components required for the assessment, particularly the trunk and neck, which were the primary focus of this study. The assessor was not allowed to move freely around the participant or observe from the front to avoid visual distraction and maintain participant concentration. We agree that this approach may limit the assessment of certain non-sagittal components (e.g., shoulder abduction, wrist deviation), and this has now been explicitly acknowledged as a limitation.

Second, the maximum RULA score was used to represent the highest RULA-derived ergonomic risk during gameplay. Based on pilot observations, participants tended to adopt and maintain a relatively stable posture once engaged in the task, particularly in the neck and trunk, with little variation over time (i.e., transitioning from baseline to a sustained posture). Therefore, the maximum score was considered appropriate to reflect the most adverse sustained posture rather than short-term changes.

While we agree that reporting typical values (e.g., median scores) could provide additional insight, this was not the primary focus of the current study. To address this concern, we have clarified the rationale in the Methods, tempered our interpretation throughout the manuscript (e.g., using "RULA-derived risk"), and added this point to the Limitations section.

Revision made Page 9/ Line 193-194
Page 10-11/ Line 222-229
Page 18/ Line 338, 340
Page 19/ Table 5
Page 21/ Line 387, 390, 392
Page 22/ Line 416-419

Reviewer's comment

3. Photogrammetry and measurement detail

o At 720×576 resolution and 3 m distance, small neck angle changes can be sensitive to pixelation/parallax. It is not clear whether anatomical markers were used (tragus, C7, T12, greater trochanter), how camera height/axis was aligned, whether lens distortion and scale calibration were performed, and how clothing was controlled.

o You report excellent reliability (ICC 0.86–0.99), but the ICC model (e.g., two way mixed, absolute agreement), sample, and 95% CI are not described.

o Actions: Add details on marker placement, palpation procedures, camera geometry/calibration, assessor training, and reliability model/CI. Include a brief limitation note on 2D sagittal estimation vs. 3D gold standards.

Author response

Thank you for these valuable comments. We have revised the Methods section to provide additional details on participant preparation, anatomical marker placement (including palpation procedures), camera setup and alignment, and the photographic measurement protocol. We have also clarified the reliability analysis by specifying the ICC model used and 95% CIs. In addition, we have included a limitation of the 2D photographic analysis.

Revision made Page 7-8/ Line 155-159, 165-171, 173-178
Page 22/ Line 421-423

Reviewer's comment

4. Pain/discomfort measurement and interpretation

o You report that all participants experienced axial discomfort; this raises the possibility of priming/demand characteristics. There is no pre condition baseline per posture, so "new onset" cannot be inferred.

o Intensity differences were non significant, likely reflecting the short exposure (20 min).

o Actions: Reframe language to "prevalence of any discomfort" rather than "pain," or consistently use "perceived discomfort" for acute, low intensity symptoms. If baseline data were collected, analyze change scores; if not, acknowledge this as a limitation. Consider reporting proportions above a minimal threshold (e.g., VAS ≥2/10) to aid clinical interpretation.

Author response

Thank you for these valuable comments. We have clarified the wording of the exclusion criteria, which already excluded participants with any resting pain. In addition, we have clarified in the Methods that participants proceeded to the next condition only after confirming that no pain or discomfort remained. We have also revised the wording in the Results and Discussion to improve clarity and more accurately reflect the nature of the reported symptoms. Furthermore, we have included the proportion of participants reporting discomfort at a minimal threshold (VAS ≥2) in Table 3 to enhance clinical interpretation.

Revision made Page 16-17/ Line 310-312, 314, 316-321
Page 17/ Table 3

Reviewer's comment

5. Order/carryover and rest interval

o You used random order with ~10 min rests. Given cognitive load and possible postural fatigue, residual effects may persist.

o Actions: Report the distribution of condition orders and (if feasible) include order/sequence as a factor/covariate (mixed models would facilitate this). Acknowledge the rest duration as a limitation if further analysis is impractical.

Author response

Thank you for these valuable comments. In the original manuscript, the description of the randomization procedure was not sufficiently detailed. We have now clarified in the Methods that the three sitting conditions were arranged using six predefined sequences to ensure a balanced distribution of order across participants. Regarding the rest interval, we have revised the Methods to clearly state that the rest period was at least 10 minutes and could be extended until any fatigue, discomfort, or related symptoms had fully resolved before proceeding to the next condition. However, we acknowledge that residual effects related to cognitive load or postural fatigue cannot be entirely excluded, and this has been added to the study limitations as suggested.

Revision made Page 10/ Line 201-205, 218-221
Page 22/ Line 415-416

Reviewer's comment
6. Device/task heterogeneity as a source of variance
o Participants used their own smartphones (size, weight, brightness), and played at their usual skill level; both can affect viewing distance, head/arm posture, and cognitive load.
o Actions: Report any available device characteristics and discuss this as a source of variability; note as a limitation. In future work, consider standardizing task segments or recording objective gameplay metrics as covariates.
Author response
Thank you for this valuable comment. We collected data on smartphone characteristics, including device weight and screen size; however, these were not reported in the original manuscript. We have now revised the manuscript to include these data in both the Methods and Results sections. In addition, we have incorporated your suggestion by acknowledging this factor as a source of variability in the study limitations and by providing recommendations for future research.
Revision made Page 9/ Line 197-198
Page 13/ Table 1
Page 22/ Line 419-421, 426-427

Reviewer's comment
7. Scope and strength of claims
o Several phrases imply long term effects ("cumulative spinal loading", "long term well being"). The study tests immediate/acute effects and does not measure loading, EMG, or long term outcomes.
o Actions: Temper to "immediate effects" and "may contribute to strain over time", noting that longitudinal confirmation is needed.
Author response
Thank you for this valuable comment. We have revised the manuscript to ensure that all statements are aligned with the scope of the study. Specifically, we have tempered the wording in the Abstract, Discussion, and Conclusion to emphasize immediate effects and to avoid overinterpretation.
Revision made Page 3/ Line 48-51
Page 22/ Line 403-409
Page 23/ Line 434-438

Reviewer's comment

8. Generalizability

o The cohort is predominantly young adult males and includes a culturally specific posture (floor cross legged).

o Actions: Expand limitations on sex/age balance, cultural context, and external validity; suggest diverse samples and varied use contexts in future research.

Author response

Thank you for this valuable comment. We have revised the limitations section to more comprehensively address factors affecting generalizability, including sex and age distribution as well as the cultural context of the sitting posture. In addition, we have incorporated your suggestions by expanding the future study section to include more diverse populations and varied usage contexts.

Revision made Page 22-23/ Line 410-413, 423-426

Minor comments (clarity, style, and presentation)

Reviewer's comment

Title & Abstract

• Consider emphasizing the acute nature and design:

o "Immediate Effects of Sitting Position on Cervical and Trunk Posture, Perceived Discomfort, and RULA Estimated Risk During Smartphone Gaming: A Randomized, Single Session Within Subject Study."

Author response

Thank you for this helpful suggestion. We have revised the title to reflect the immediate nature of the study as recommended. Minor wording adjustments were also made to ensure consistency with the terminology used in the main text and to improve conciseness.

Revision made Page 1/ Line 1-3 (Title) and minor wording adjustments through the main text.

Reviewer's comment

• In the abstract, briefly state: design, tools (2D photogrammetry, VAS, RULA from sagittal observation, maximum score), and provide effect sizes for main outcomes alongside p values.

Author response

Thank you for this helpful suggestion. We have revised the abstract to include additional methodological details and key results, including the study design, measurement tools, and effect sizes, as recommended. These revisions were made while ensuring compliance with the journal's formatting requirements and word limit.

Revision made Page 2/ Line 30, 33-36, 38-43

Methods

Reviewer's comment

• Randomization: Describe allocation (e.g., block vs simple), concealment, and order balancing.

Author response

Thank you for this helpful suggestion. We have clarified the randomization process and provided additional details on sequence allocation in the Methods section under the Study procedure subsection.

Revision made Page 10/ Line 201-205

Reviewer's comment

• Chair/floor setup: Specify floor surface (hard/soft, cushion), any forearm support (lap/thighs), and chair dimensions.

Author response

Thank you for this suggestion. We have revised the Methods section to include additional details regarding the chair characteristics and the floor surface used for the cross-legged sitting condition.

Revision made Page 10/ Line 205-211

Reviewer's comment

• Assessor blinding: Clarify whether the RULA assessor was blinded to hypotheses (condition blinding is not feasible).

Author response

Thank you for this helpful suggestion. We acknowledge the potential for assessor bias, and blinding to sitting conditions was not feasible due to the nature of the study protocol. However, the assessor underwent prior training and followed standardized RULA scoring procedures to ensure consistency and minimize potential bias. We provided additional details in the Methods section under the Study procedure subsection.

Revision made Page 10-11/ Line 222-229

Reviewer's comment

• Power analysis: State assumed correlation among repeated measures (if any) and exact G*Power settings.

Author response

Thank you for this helpful suggestion. We have revised the Methods section (Sample Size Estimation subsection) to provide additional details on the G*Power analysis, including the assumptions and parameters used for the sample size calculation.

Revision made Page 7/ Line 140-141

Reviewer's comment

• Terminology: Use "perceived discomfort" for acute symptoms to avoid implying clinical pain.

Author response

Thank you for this suggestion. We have carefully reviewed the manuscript and revised the terminology accordingly, replacing "pain" with "perceived discomfort" where appropriate to better reflect the acute nature of the symptoms.

Revision made Page 1 / Line 1
Page 3/ Line 46
Page 16/ Line 310-317
Page 20/ Line 378

Results

Reviewer's comment

• For interactions, present simple effects (differences between positions at each time point with adjustment).

Author response

Thank you for this suggestion. We have clarified the reporting of interaction effects by describing the simple effects and presenting pairwise comparisons between sitting positions at each time point, as detailed in the supplementary tables.

Revision made S1 and S2 Tables

Reviewer's comment

• Provide estimated marginal means (95% CI) for angles and RULA.

Author response

Thank you for this helpful suggestion. Estimated marginal means with 95% confidence intervals for cervical and trunk angles were already reported in Table 2 and the corresponding pairwise comparisons in S1 and S2 Tables. For RULA outcomes, we have revised Table 5 to include 95% confidence intervals as requested.

Revision made Page 15/ Table 2
Page 19/ Table 5
S1 and S2 Tables

Reviewer's comment

• Figures: Consider adding spaghetti plots (light lines per participant) with overlaid means ± SD/CI to show within subject variance.

Author response

Thank you for this helpful suggestion. We have added spaghetti plots with individual trajectories and overlaid mean ± 95% CI for both cervical and trunk angles across all three sitting conditions, presented in the Supplementary Figures (S2–S7).

Revision made S2-S7 Figures

Reviewer's comment
• Ensure correct phrasing ("not significant," not "not significance").
Author response
Thank you for this helpful comment. We have corrected the phrasing to ensure the appropriate use of "not significant".
Revision made Page 14/ Line 288

Tables/Figures
Reviewer's comment
• Table 2: Ensure df and p values match the final analysis; keep units and capitalization consistent.
Author response
Thank you for this helpful comment. We have carefully reviewed and corrected Table 2 to ensure that all degrees of freedom and p-values are consistent with the final analyses reported in the main text. Units and capitalization have also been standardized throughout the table.
Revision made Page 15/ Table 2

Reviewer's comment
• Tables 3–5: Add effect sizes (Kendall's W, r). Clarify post hoc corrections in footnotes.
Author response
Thank you for this helpful suggestion. We have revised Tables 3-5 to include effect sizes (Kendall's W) and have clarified the post hoc corrections in the corresponding table footnotes. In addition, the statistical analysis subsection in the Methods section has been updated.
Revision made Page 17-19/ Table 3-5
Page 12/ Line 254-255

Reviewer's comment
• Ensure all figure axes are labelled with units; include clear legends.
Author response
Thank you for this helpful comment. We have reviewed all figures and ensured that axes are appropriately labeled with units and that legends are clear and consistent throughout.
Revision made All figures

Reviewer's comment
• Correct typos: e.g., "supportting" → "supporting" (line 366).
Author response
Thank you for this helpful comment. We have corrected typographical errors.
Revision made Page 23/ Line 437

Discussion

Reviewer's comment

• When citing comparable studies, distinguish task type (texting, video, gaming), duration, and support conditions to explain differences in risk levels.

Author response

Thank you for this helpful comment. We have revised the Discussion section to more clearly explain differences in ergonomic risk levels.

Revision made Page 21/ Line 393-398

Reviewer's comment

• Add an explicit limitations paragraph: single plane RULA, 2D angles, order effects, device heterogeneity, short exposure, male dominant sample.

Author response

Thank you for this helpful suggestion. We have included a detailed limitations paragraph addressing all the points raised.

Revision made Page 22/ Line 410-423

Reviewer 2

Reviewer's comment

1. Lines 145-158: I propose adding figures illustrating the assessed cervical and trunk parameters.

Author response

Thank you for this helpful suggestion. We have added a figure illustrating the measurement of cervical and trunk angles (neck flexion and trunk angle) to align with the descriptions provided in the main text.

Revision made Figure 2

Reviewer's comment

2. Line 173: "All assesors…" Who were the assesors and what was their experience?

Author response

Thank you for this helpful comment. We have revised the Methods section to include additional details regarding the assessors and their experience.

Revision made Page 7/ Line 146-152

Reviewer's comment

3. Lines 222-223: "(…), 7 participants did not meet the inclusion and exclusion criteria". I don't understand the phrasing that the participants did not meet the exclusion criteria.

Author response

Thank you for this helpful comment. We apologize for the ambiguity in the wording, which may have caused confusion. The seven participants met the exclusion criteria and were excluded accordingly. This has been clarified in the revised Results section.

Revision made Page 12/ Line 257-258

Reviewer's comment

4. Table 1: Where can I find the data related to the female participants?

Author response

Thank you for this helpful comment. We have revised Table 1 to include data for both male and female participants to provide a complete description of the sample. All other descriptive variables are reported for the total sample (n = 41); however, the previous omission of female data in the sex category may have caused confusion, which has now been corrected.

Revision made Page 13/ Table 1

Reviewer's comment

5. I suggest adding photos of the sitting positions.

Author response

Thank you for this helpful suggestion. We have added photographs of the sitting positions to the Supplementary Materials.

Revision made S1 Figure

Reviewer 3

Reviewer's comment

Reviewer #3: This is a well-presented report addressing an important issue. At this stage, however, I would have one key issue that do not allow a comprehensive review. The authors state that they employed 'a laboratory-based within-subject repeated-measures experimental design' but do not seem to utilise any reporting guidelines. Please address this issue and, ideally, amend the report with reporting guidelines elements (such as a flow chart and a checklist). A flowchart is presented separately, but it is not clear where it is placed in the text. Does the study design comply with the CONSORT2020 / SPIRIT fuidelines?

Author response

Thank you for this important comment. We would like to clarify that this study was reported in accordance with the STROBE guidelines. As this study does not involve a randomized controlled trial design, the CONSORT guidelines were not applicable, and as it is not a study protocol, the SPIRIT guidelines were also not appropriate. Therefore, we considered STROBE to be the most suitable reporting guideline for this study.

We note that the STROBE checklist had already been completed and submitted in the initial submission; however, this was not explicitly stated in the main manuscript, which may have led to this concern. In addition, the participant flow diagram was already presented in Figure 1, but it was previously cited only in the Results section.

In the revised manuscript, we have now explicitly stated the reporting guideline used in the Study Design section and appropriately referenced the participant flow (Fig 1) within this section. We have also updated the STROBE checklist to ensure that page numbers correspond accurately to the revised version of the manuscript.

**Revision made**	Page 6/ Line 122-124
	Figure 1
	Supplementary Materials (STROBE checklist)
