# Supplementary material for: Immediate effects of sitting position on cervical and trunk posture, perceived discomfort, and RULA-based ergonomic risk during smartphone gaming: A randomized within-subject study
Source: PLoS One. 2026 Jul 21;21(7):e0354360. doi: 10.1371/journal.pone.0354360 (PMC13387541; doi:10.1371/journal.pone.0354360)
Supplement: Peer review history

# Peer review: Decision Letter 0

**DOI:** 10.1371/journal.pone.0354360.r001

**Authors:** Mshari Alghadier

31 Mar 2026

PONE-D-26-01989

Effect of Sitting Position on Cervical and Trunk Posture, Musculoskeletal Pain, and Ergonomic Risk During Smartphone Gaming

PLOS One

Dear Dr. Yodchaisarn,

Thank you for submitting your manuscript to PLOS ONE. After careful consideration, we feel that it has merit but does not fully meet PLOS ONE’s publication criteria as it currently stands. Therefore, we invite you to submit a revised version of the manuscript that addresses the points raised during the review process.

Please submit your revised manuscript by May 15 2026 11:59PM. If you will need more time than this to complete your revisions, please reply to this message or contact the journal office at plosone@plos.org. When you're ready to submit your revision, log on to https://www.editorialmanager.com/pone/ and select the 'Submissions Needing Revision' folder to locate your manuscript file.

Please include the following items when submitting your revised manuscript:

- A letter that responds to each point raised by the academic editor and reviewer(s). You should upload this letter as a separate file labeled 'Response to Reviewers'.
- A marked-up copy of your manuscript that highlights changes made to the original version. You should upload this as a separate file labeled 'Revised Manuscript with Track Changes'.
- An unmarked version of your revised paper without tracked changes. You should upload this as a separate file labeled 'Manuscript'.

If you would like to make changes to your financial disclosure, please include your updated statement in your cover letter. Guidelines for resubmitting your figure files are available below the reviewer comments at the end of this letter.

If applicable, we recommend that you deposit your laboratory protocols in protocols.io to enhance the reproducibility of your results. Protocols.io assigns your protocol its own identifier (DOI) so that it can be cited independently in the future. For instructions see: https://journals.plos.org/plosone/s/submission-guidelines#loc-laboratory-protocols. Additionally, PLOS ONE offers an option for publishing peer-reviewed Lab Protocol articles, which describe protocols hosted on protocols.io. Read more information on sharing protocols at https://plos.org/protocols?utm_medium=editorial-email&utm_source=authorletters&utm_campaign=protocols.

As the corresponding author, your ORCID iD is verified in the submission system and will appear in the published article. PLOS supports the use of ORCID, and we encourage all coauthors to register for an ORCID iD and use it as well. Please encourage your coauthors to verify their ORCID iD within the submission system before final acceptance, as unverified ORCID iDs will not appear in the published article. *Only*  the individual author can complete the verification step; PLOS staff *cannot*  verify ORCID iDs on behalf of authors.

We look forward to receiving your revised manuscript.

Kind regards,

Mshari Alghadier

Academic Editor

PLOS One

**Journal Requirements:**

1. When submitting your revision, we need you to address these additional requirements.

Please ensure that your manuscript meets PLOS ONE's style requirements, including those for file naming. The PLOS ONE style templates can be found at

https://journals.plos.org/plosone/s/file?id=wjVg/PLOSOne_formatting_sample_main_body.pdf and

https://journals.plos.org/plosone/s/file?id=ba62/PLOSOne_formatting_sample_title_authors_affiliations.pdf

2. We note that you have indicated that there are restrictions to data sharing for this study. For studies involving human research participant data or other sensitive data, we encourage authors to share de-identified or anonymized data. However, when data cannot be publicly shared for ethical reasons, we allow authors to make their data sets available upon request. For information on unacceptable data access restrictions, please see http://journals.plos.org/plosone/s/data-availability#loc-unacceptable-data-access-restrictions.

Before we proceed with your manuscript, please address the following prompts:

a) If there are ethical or legal restrictions on sharing a de-identified data set, please explain them in detail (e.g., data contain potentially identifying or sensitive patient information, data are owned by a third-party organization, etc.) and who has imposed them (e.g., a Research Ethics Committee or Institutional Review Board, etc.). Please also provide contact information for a data access committee, ethics committee, or other institutional body to which data requests may be sent.

b) If there are no restrictions, please upload the minimal anonymized data set necessary to replicate your study findings to a stable, public repository and provide us with the relevant URLs, DOIs, or accession numbers. Please see http://www.bmj.com/content/340/bmj.c181.long for guidelines on how to de-identify and prepare clinical data for publication. For a list of recommended repositories, please see https://journals.plos.org/plosone/s/recommended-repositories. You also have the option of uploading the data as Supporting Information files, but we would recommend depositing data directly to a data repository if possible.

Please update your Data Availability statement in the submission form accordingly.

3. If the reviewer comments include a recommendation to cite specific previously published works, please review and evaluate these publications to determine whether they are relevant and should be cited. There is no requirement to cite these works unless the editor has indicated otherwise.

[Note: HTML markup is below. Please do not edit.]

Reviewers' comments:

Reviewer's Responses to Questions

**Comments to the Author**

1. Is the manuscript technically sound, and do the data support the conclusions?

The manuscript must describe a technically sound piece of scientific research with data that supports the conclusions. Experiments must have been conducted rigorously, with appropriate controls, replication, and sample sizes. The conclusions must be drawn appropriately based on the data presented.

Reviewer #1: Partly

Reviewer #2: Yes

Reviewer #3: Partly

**********

2. Has the statistical analysis been performed appropriately and rigorously?

Reviewer #1: I Don't Know

Reviewer #2: Yes

Reviewer #3: Yes

**********

3. Have the authors made all data underlying the findings in their manuscript fully available?

The PLOS Data policy requires authors to make all data underlying the findings described in their manuscript fully available without restriction, with rare exception (please refer to the Data Availability Statement in the manuscript PDF file). The data should be provided as part of the manuscript or its supporting information, or deposited to a public repository. For example, in addition to summary statistics, the data points behind means, medians and variance measures should be available. If there are restrictions on publicly sharing data—e.g. participant privacy or use of data from a third party—those must be specified.

Reviewer #1: Yes

Reviewer #2: Yes

Reviewer #3: Yes

**********

4. Is the manuscript presented in an intelligible fashion and written in standard English?

PLOS ONE does not copyedit accepted manuscripts, so the language in submitted articles must be clear, correct, and unambiguous. Any typographical or grammatical errors should be corrected at revision, so please note any specific errors here.

Reviewer #1: Yes

Reviewer #2: Yes

Reviewer #3: Yes

**********

5. Review Comments to the Author

Please use the space provided to explain your answers to the questions above. You may also include additional comments for the author, including concerns about dual publication, research ethics, or publication ethics. (Please upload your review as an attachment if it exceeds 20,000 characters)

**Reviewer #1:**  Reviewer Report

Manuscript title: Effect of Sitting Position on Cervical and Trunk Posture, Musculoskeletal Pain, and Ergonomic Risk During Smartphone Gaming

Overall recommendation: Major revision

1) Summary (what the study did and found)

This is a single session, randomized within subject study (N=41; young adults) examining the acute effects of three sitting positions—chair with backrest, chair without backrest, and cross legged floor sitting—on cervical/trunk posture (2D photogrammetry at 0–20 min), perceived musculoskeletal discomfort (post session location and VAS intensity), and ergonomic risk (RULA, sagittal observation; maximum score across 20 min). Key findings: (i) significant position × time interactions for neck and trunk angles; (ii) cross legged sitting produced the greatest neck flexion and highest prevalence of neck discomfort; (iii) RULA scores/levels were higher in cross legged sitting than both chair conditions. The work addresses a practical and under studied posture (floor cross legged) with direct ergonomic implications for mobile gaming.

2) Major comments (substantive issues to address)

1. Statistical reporting and analysis

o Please can you check if your repeated measures ANOVA degrees of freedom align with N=41?

2. RULA scoring validity from sagittal only assessment

o RULA requires inputs that can involve non sagittal planes (e.g., shoulder abduction, wrist deviation). Scoring from sagittal view alone can misclassify risk.

o You also use the maximum observed RULA as the representative score; this may overstate risk relative to typical postures.

o Actions: Clearly state how full RULA items were operationalized from a single view; acknowledge limitations; temper claims to “RULA derived risk from sagittal observation”; consider also reporting a typical/mid session or median RULA score in the Results (or a sensitivity analysis) alongside the maximum.

3. Photogrammetry and measurement detail

o At 720×576 resolution and 3 m distance, small neck angle changes can be sensitive to pixelation/parallax. It is not clear whether anatomical markers were used (tragus, C7, T12, greater trochanter), how camera height/axis was aligned, whether lens distortion and scale calibration were performed, and how clothing was controlled.

o You report excellent reliability (ICC 0.86–0.99), but the ICC model (e.g., two way mixed, absolute agreement), sample, and 95% CI are not described.

o Actions: Add details on marker placement, palpation procedures, camera geometry/calibration, assessor training, and reliability model/CI. Include a brief limitations note on 2D sagittal estimation vs. 3D gold standards.

4. Pain/discomfort measurement and interpretation

o You report that all participants experienced axial discomfort; this raises the possibility of priming/demand characteristics. There is no pre condition baseline per posture, so “new onset” cannot be inferred.

o Intensity differences were non significant, likely reflecting the short exposure (20 min).

o Actions: Reframe language to “prevalence of any discomfort” rather than “pain,” or consistently use “perceived discomfort” for acute, low intensity symptoms. If baseline data were collected, analyze change scores; if not, acknowledge this as a limitation. Consider reporting proportions above a minimal threshold (e.g., VAS ≥2/10) to aid clinical interpretation.

5. Order/carryover and rest interval

o You used random order with ~10 min rests. Given cognitive load and possible postural fatigue, residual effects may persist.

o Actions: Report the distribution of condition orders and (if feasible) include order/sequence as a factor/covariate (mixed models would facilitate this). Acknowledge the rest duration as a limitation if further analysis is impractical.

6. Device/task heterogeneity as a source of variance

o Participants used their own smartphones (size, weight, brightness), and played at their usual skill level; both can affect viewing distance, head/arm posture, and cognitive load.

o Actions: Report any available device characteristics and discuss this as a source of variability; note as a limitation. In future work, consider standardizing task segments or recording objective gameplay metrics as covariates.

7. Scope and strength of claims

o Several phrases imply long term effects (“cumulative spinal loading”, “long term well being”). The study tests immediate/acute effects and does not measure loading, EMG, or long term outcomes.

o Actions: Temper to “immediate effects” and “may contribute to strain over time”, noting that longitudinal confirmation is needed.

8. Generalizability

o The cohort is predominantly young adult males and includes a culturally specific posture (floor cross legged).

o Actions: Expand limitations on sex/age balance, cultural context, and external validity; suggest diverse samples and varied use contexts in future research.

3) Minor comments (clarity, style, and presentation)

Title & Abstract

• Consider emphasizing the acute nature and design:

o “Immediate Effects of Sitting Position on Cervical and Trunk Posture, Perceived Discomfort, and RULA Estimated Risk During Smartphone Gaming: A Randomized, Single Session Within Subject Study.”

• In the abstract, briefly state: design, tools (2D photogrammetry, VAS, RULA from sagittal observation, maximum score), and provide effect sizes for main outcomes alongside p values.

Methods

• Randomization: Describe allocation (e.g., block vs simple), concealment, and order balancing.

• Chair/floor setup: Specify floor surface (hard/soft, cushion), any forearm support (lap/thighs), and chair dimensions.

• Assessor blinding: Clarify whether the RULA assessor was blinded to hypotheses (condition blinding is not feasible).

• Power analysis: State assumed correlation among repeated measures (if any) and exact G*Power settings.

• Terminology: Use “perceived discomfort” for acute symptoms to avoid implying clinical pain.

Results

• For interactions, present simple effects (differences between positions at each time point with adjustment).

• Provide estimated marginal means (95% CI) for angles and RULA.

• Figures: Consider adding spaghetti plots (light lines per participant) with overlaid means ± SD/CI to show within subject variance.

• Ensure correct phrasing (“not significant,” not “not significance”).

Tables/Figures

• Table 2: Ensure df and p values match the final analysis; keep units and capitalization consistent.

• Tables 3–5: Add effect sizes (Kendall’s W, r). Clarify post hoc corrections in footnotes.

• Ensure all figure axes are labelled with units; include clear legends.

• Correct typos: e.g., “supportting” → “supporting” (line 366).

Discussion

• When citing comparable studies, distinguish task type (texting, video, gaming), duration, and support conditions to explain differences in risk levels.

• Add an explicit limitations paragraph: single plane RULA, 2D angles, order effects, device heterogeneity, short exposure, male dominant sample.

**Reviewer #2:**  Dear Authors

I enjoyed reading your paper. The study concerns an important problem of effect of sitting position on cervical and trunk posture, musculoskeletal pain, and ergonomic risk during smartphone gaming. The study aimed to analyze the effects of three sitting positions, including chair sitting with and without a backrest and cross-legged sitting on the floor, on cervical and trunk postures, musculoskeletal pain or discomfort, and ergonomic risk during smartphone gaming among young adults.

However, I have a couple of minor requests to be revised as stated below.

Comments:

1. Lines 145-158: I propose adding figures illustrating the assessed cervical and trunk parameters.

2. Line 173: “All assesors…” Who were the assesors and what was their experience?

3. Lines 222-223: “(…), 7 participants did not meet the inclusion and exclusion criteria”. I don’t understand the phrasing that the participants did not meet the exclusion criteria.

4. Table 1: Where can I find the data related to the female participants?

5. I suggest adding photos of the sitting positions.

**Reviewer #3:**  This is a well-presented report addressing an important issue. At this stage, however, I would have one key issue that do not allow a comprehensive review. The authors state that they employed ‘a laboratory-based within-subject repeated-measures experimental design’ but do not seem to utilise any reporting guidelines. Please address this issue and, ideally, amend the report with reporting guidelines elements (such as a flow chart and a checklist). A flowchart is presented separately, but it is not clear where it is placed in the text. Does the study design comply with the CONSORT2020 / SPIRIT fuidelines?

**********

6. PLOS authors have the option to publish the peer review history of their article (what does this mean?). If published, this will include your full peer review and any attached files.

If you choose “no”, your identity will remain anonymous but your review may still be made public.

**Do you want your identity to be public for this peer review?**  For information about this choice, including consent withdrawal, please see our Privacy Policy.

Reviewer #1: **Yes:**  Nonso Asouzu

Reviewer #2: No

Reviewer #3: **Yes:**  Maciej Płaszewski

**********

[NOTE: If reviewer comments were submitted as an attachment file, they will be attached to this email and accessible via the submission site. Please log into your account, locate the manuscript record, and check for the action link "View Attachments". If this link does not appear, there are no attachment files.]

To ensure your figures meet our technical requirements, please review our figure guidelines: https://journals.plos.org/plosone/s/figures

You may also use PLOS’s free figure tool, NAAS, to help you prepare publication quality figures: https://journals.plos.org/plosone/s/figures#loc-tools-for-figure-preparation.

NAAS will assess whether your figures meet our technical requirements by comparing each figure against our figure specifications.

Attachment Submitted filename: Review.pdf

# Author comment: Author response to Decision Letter 1

**DOI:** 10.1371/journal.pone.0354360.r002

12 May 2026

Please find attached the detailed responses to the reviewers. All comments have been carefully addressed in the revised manuscript and supplementary files.

Attachment Submitted filename: Response to reviewer.pdf

# Peer review: Decision Letter 1

**DOI:** 10.1371/journal.pone.0354360.r003

**Authors:** Mshari Alghadier

8 Jul 2026

Immediate effects of sitting position on cervical and trunk posture, perceived discomfort, and RULA-based ergonomic risk during smartphone gaming: a randomized within-subject study

PONE-D-26-01989R1

Dear Dr. Yodchaisarn,

We’re pleased to inform you that your manuscript has been judged scientifically suitable for publication and will be formally accepted for publication once it meets all outstanding technical requirements.

Within one week, you’ll receive an e-mail detailing the required amendments. When these have been addressed, you’ll receive a formal acceptance letter and your manuscript will be scheduled for publication.

An invoice will be generated when your article is formally accepted. Please note, if your institution has a publishing partnership with PLOS and your article meets the relevant criteria, all or part of your publication costs will be covered. Please make sure your user information is up-to-date by logging into Editorial Manager at Editorial Manager® and clicking the ‘Update My Information' link at the top of the page. For questions related to billing, please contact billing support.

If your institution or institutions have a press office, please notify them about your upcoming paper to help maximize its impact. If they’ll be preparing press materials, please inform our press team as soon as possible -- no later than 48 hours after receiving the formal acceptance. Your manuscript will remain under strict press embargo until 2 pm Eastern Time on the date of publication. For more information, please contact onepress@plos.org.

Kind regards,

Mshari Alghadier

Academic Editor

PLOS One

Additional Editor Comments (optional):

Reviewers' comments:

Reviewer's Responses to Questions

**Comments to the Author**

1. If the authors have adequately addressed your comments raised in a previous round of review and you feel that this manuscript is now acceptable for publication, you may indicate that here to bypass the “Comments to the Author” section, enter your conflict of interest statement in the “Confidential to Editor” section, and submit your "Accept" recommendation.

Reviewer #2: All comments have been addressed

Reviewer #3: All comments have been addressed

**********

2. Is the manuscript technically sound, and do the data support the conclusions?

The manuscript must describe a technically sound piece of scientific research with data that supports the conclusions. Experiments must have been conducted rigorously, with appropriate controls, replication, and sample sizes. The conclusions must be drawn appropriately based on the data presented.

Reviewer #2: Yes

Reviewer #3: Yes

**********

3. Has the statistical analysis been performed appropriately and rigorously?

Reviewer #2: Yes

Reviewer #3: Yes

**********

4. Have the authors made all data underlying the findings in their manuscript fully available?

The PLOS Data policy requires authors to make all data underlying the findings described in their manuscript fully available without restriction, with rare exception (please refer to the Data Availability Statement in the manuscript PDF file). The data should be provided as part of the manuscript or its supporting information, or deposited to a public repository. For example, in addition to summary statistics, the data points behind means, medians and variance measures should be available. If there are restrictions on publicly sharing data—e.g. participant privacy or use of data from a third party—those must be specified.

Reviewer #2: Yes

Reviewer #3: Yes

**********

5. Is the manuscript presented in an intelligible fashion and written in standard English?

PLOS ONE does not copyedit accepted manuscripts, so the language in submitted articles must be clear, correct, and unambiguous. Any typographical or grammatical errors should be corrected at revision, so please note any specific errors here.

Reviewer #2: Yes

Reviewer #3: Yes

**********

6. Review Comments to the Author

Please use the space provided to explain your answers to the questions above. You may also include additional comments for the author, including concerns about dual publication, research ethics, or publication ethics. (Please upload your review as an attachment if it exceeds 20,000 characters)

Reviewer #2: I would like to thank the Authors for addressing all the comments. After completing the missing elements and providing a more detailed description of the research process, the manuscript can be accepted for publication. The topic of discomfort in the cervical spine and its curvature during prolonged sitting is both timely and highly important.

Reviewer #3: Dear Authors,

Thank you for your amendments. I have no further issues regarding your submission.

I congratulate your paper.

**********

7. PLOS authors have the option to publish the peer review history of their article (what does this mean?). If published, this will include your full peer review and any attached files.

If you choose “no”, your identity will remain anonymous but your review may still be made public.

**Do you want your identity to be public for this peer review?**  For information about this choice, including consent withdrawal, please see our Privacy Policy.

Reviewer #2: No

Reviewer #3: **Yes:**  Maciej Płaszewski

**********

# Editor report: Acceptance letter

**DOI:** 10.1371/journal.pone.0354360.r004

**Authors:** Mshari Alghadier

PONE-D-26-01989R1

PLOS One

Dear Dr. Yodchaisarn,

I'm pleased to inform you that your manuscript has been deemed suitable for publication in PLOS One. Congratulations! Your manuscript is now being handed over to our production team.

At this stage, our production department will prepare your paper for publication. This includes ensuring the following:

* All references, tables, and figures are properly cited

* All relevant supporting information is included in the manuscript submission,

* There are no issues that prevent the paper from being properly typeset

You will receive further instructions from the production team, including instructions on how to review your proof when it is ready. Please keep in mind that we are working through a large volume of accepted articles, so please give us a few days to review your paper and let you know the next and final steps.

Lastly, if your institution or institutions have a press office, please let them know about your upcoming paper now to help maximize its impact. If they'll be preparing press materials, please inform our press team within the next 48 hours. Your manuscript will remain under strict press embargo until 2 pm Eastern Time on the date of publication. For more information, please contact onepress@plos.org.

You will receive an invoice from PLOS for your publication fee after your manuscript has reached the completed accept phase. If you receive an email requesting payment before acceptance or for any other service, this may be a phishing scheme. Learn how to identify phishing emails and protect your accounts at https://explore.plos.org/phishing.

If we can help with anything else, please email us at customercare@plos.org.

Thank you for submitting your work to PLOS ONE and supporting open access.

Kind regards,

PLOS ONE Editorial Office Staff

on behalf of

Dr. Mshari Alghadier

Academic Editor

PLOS One
